# Genomic analysis of the carboxylesterase family in the salmon louse (*Lepeophtheirus salmonis*)

**DOI:** 10.1016/j.cbpc.2021.109095

**Published:** 2021-10

**Authors:** Claudia Tschesche, Michaël Bekaert, Joseph L. Humble, James E. Bron, Armin Sturm

**Affiliations:** Institute of Aquaculture, Faculty of Natural Sciences, University of Stirling, Stirling FK9 4LA, United Kingdom

**Keywords:** Salmon lice, Resistance, Carboxylesterase, Deltamethrin, Emamectin benzoate

## Abstract

The pyrethroid deltamethrin and the macrocyclic lactone emamectin benzoate (EMB) are used to treat infestations of farmed salmon by parasitic salmon lice, *Lepeophtheirus salmonis*. While the efficacy of both compounds against Atlantic populations of the parasite has decreased as a result of the evolution of resistance, the molecular mechanisms of drug resistance in *L. salmonis* are currently not fully understood. The functionally diverse carboxylesterases (CaE) family includes members involved in pesticide resistance phenotypes of terrestrial arthropods. The present study had the objective to characterize the CaE family in *L. salmonis* and assess its role in drug resistance. *L*. *salmonis* CaE homologues were identified by homology searches in the parasite's transcriptome and genome. The transcript expression of CaEs predicted to be catalytically competent was studied using quantitative reverse-transcription PCR in drug susceptible and multi-resistant *L. salmonis*. The above strategy led to the identification of 21 CaEs genes/pseudogenes. Phylogenetic analyses assigned 13 CaEs to clades involved in neurodevelopmental signaling and cell adhesion, while three sequences were predicted to encode secreted enzymes. Ten CaEs were identified as being potentially catalytically competent. Transcript expression of acetylcholinesterase (*ace1b*) was significantly increased in multi-resistant lice compared to drug-susceptible *L. salmonis*, with transcript abundance further increased in preadult-II females following EMB exposure. In summary, results from the present study demonstrate that *L. salmonis* possesses fewer CaE gene family members than most arthropods characterized so far. Drug resistance in *L. salmonis* was associated with overexpression of *ace1b*.

## Highlights

• 21 genes/pseudogenes coding for carboxylesterases (CaEs) identified in L. *salmonis*
• Differential expression of *ace1b* between drug susceptible and resistant L. *salmonis*
• Regulation of transcript expression of *ace1b* following sublethal EMB concentrations
• No evidence for key role of CaEs in resistance of L. *salmonis* to DTM and EMB

## Graphical abstract

**Figure:**
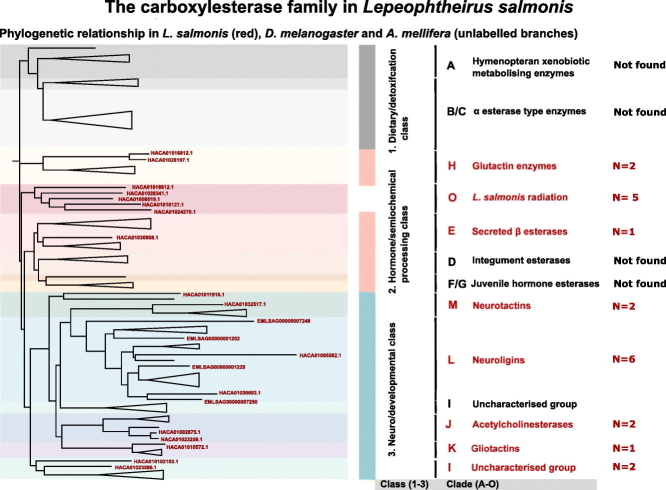

## 1 Introduction

Sea lice of the family Caligidae (Copepoda) are ectoparasites of marine fish that feed on the mucus, skin, and blood of their hosts (Boxaspen, 2006). Depending on the severity of infections, sea lice can cause adverse effects in their fish hosts that include skin lesions, which are associated with a high risk of secondary infections, as well as osmoregulatory dysfunction, immunosuppression, increased stress, and reduced food conversion and growth rates (Grimnes and Jakobsen, 1996; Wootten et al., 1982). In 2018 the global costs of sea lice infestations to the salmon industry were estimated to exceed US $873 million/£700 million (Brooker et al., 2018b), comprising costs for prevention and treatments and, to a lesser extent, losses in production. In the Northern hemisphere, the salmon louse *Lepeophtheirus salmonis* (Krøyer, 1837) is the major caligid species infecting salmonid fish (Costello, 2009). At salmon production sites, sea lice are controlled using integrated pest management strategies (IPM) combining veterinary drug treatments (Burridge et al., 2010) with a range of non-medicinal control approaches, which include mechanical and thermal delousing (reviewed in Holan et al., 2017) as well as the deployment of different species of cleaner fish that remove caligids from farmed salmon (Brooker et al., 2018a), as well as. Pharmaceuticals used for the control of sea lice are administered either orally as feed additives or topically as bath treatments. In-feed treatments include the macrocyclic lactone emamectin benzoate (EMB) and different benzoylureas, while bath treatments include the organophosphate azamethiphos, the disinfectant hydrogen peroxide and the pyrethroids cypermethrin and deltamethrin (DTM) (Helgesen et al., 2019).

The continual use of a limited range of chemotherapeutants in pest control, with insufficient rotation between products of dissimilar mode of action, can lead to the evolution of resistance (Tabashnik et al., 2014). In treatment of *L. salmonis* infections, losses of efficacy have been reported for most available anti-parasitic drugs (Helgesen et al., 2019). In terrestrial arthropods, insecticide resistance most commonly involves one or both of two main molecular mechanisms. Resistance can result from mutations in genes coding for proteins constituting target sites of the pesticide (Williamson et al., 1993), or it can be based on enhanced detoxification by enzymes that break-down or sequester the pesticide (Ranson et al., 2002). Metabolic resistance typically involves members of large gene families with roles in detoxification, such as the carboxylesterases (CaEs), cytochrome P450s (CYPs), glutathione-S-transferases (GSTs), and ATP binding cassette (ABC) proteins.

Recent studies have identified molecular changes associated with pesticide resistance in *L. salmonis*. *L*. *salmonis* resistance to the organophosphate azamethiphos is primarily caused by a non-synonymous target-site mutation in the gene coding for acetylcholinesterase (AChE) (Kaur et al., 2015b). Resistance of *L. salmonis* to the non-specific oxidant hydrogen peroxide has been linked to induction of catalase gene expression and enzymatic activity, as well as differential expression of five candidate genes including an aquaporin (Agusti-Ridaura et al., 2020). DTM resistance has been shown to be mainly inherited maternally and to be associated with mutations in the mitochondrial genome (mtDNA) (Carmona-Antoñanzas et al., 2017). In addition, a sodium channel mutation potentially further contributing to DTM resistance has been identified (Carmona-Antoñanzas et al., 2019). EMB resistance has been linked to selective sweeps, with the genes under selection awaiting to be identified (Besnier et al., 2014). While the genomic complement of ABC transporters and CYPs in *L. salmonis* has been described (Carmona-Antoñanzas et al., 2015; Humble et al., 2019), existing studies do not provide evidence for an involvement of overexpression of members of these gene families in drug resistance in *L. salmonis* (Carmichael et al., 2013; Humble et al., 2019; Sutherland et al., 2015).

Esterases are a large group of metabolic enzymes that can be involved in resistance of arthropod pests to a wide range of chemical control agents, including pyrethroids and organophosphate esters (reviewed in Li et al., 2007). Most esterases involved in pesticide metabolism belong to the CaE gene family (Pfam PF00135 domain), a branch within the α/β-hydrolase fold superfamily (Pfam PF00561 domain) (Punta et al., 2012). The CaE family is functionally diverse. It comprises highly specialized enzymes acting on specific substrates, as well as less-selective enzymes with broad ranges of substrates, and catalytically inactive members with different roles including neurodevelopmental signaling or surface recognition (Oakeshott et al., 2005). Catalytically active CaEs possess a catalytic triad with a nucleophilic residue (serine (Ser), cysteine (Cys), or aspartate (Asp)), an acidic residue (glutamate (Glu) or Asp), and a histidine (His) residue (Myers et al., 1988). Some catalytically active CaEs catalyze the hydrolysis of ester pesticides, such as pyrethroids and organophosphates, into their corresponding acid and alcohol metabolites, which usually show low toxicity and are excreted readily. Furthermore, catalytically active CaEs have been shown to mediate resistance by sequestering ester and non-ester pesticides, impairing interactions with their toxicological target-sites (Hemingway, 2000). Esterase-mediated sequestration has, for example, been suggested to play an important role in resistance to the macrocyclic lactone spinosad (Herron et al., 2014).

In terrestrial arthropods, different molecular mechanisms of insecticide resistance involving esterases have been described (reviewed by Hemingway, 2000). Pesticide resistance can be based on the increased expression of esterases following gene amplification (Field and Devonshire, 1998; Rooker et al., 1996). Furthermore, single point mutations around the CaEs active site have been shown to induce organophosphate resistance by endowing the mutant enzyme with the ability to hydrolyze the pesticide (Campbell et al., 1998; Claudianos et al., 1999; Newcomb et al., 1997). In addition, constitutive upregulation of CaE gene expression has been implicated in pesticide resistance in several insect species (Zhu and Luttrell, 2015).

In *L. salmonis*, little is known about the CaE family and its potential roles in drug resistance. The aim of the present study was to identify members of the CaE family in *L. salmonis* and characterize their potential roles in resistance of the parasite to salmon delousing agents. Sequences encoding *L. salmonis* CaEs were isolated by homology searches of transcriptome and genome assemblies and annotated. Subsequently, CaE sequences were analyzed in silico to identify proteins that are predicted to be catalytically competent and thus, have the potential to mediate pesticide resistance by hydrolysis or sequestration. Finally, potentially catalytically active CaEs were characterized regarding their transcript expression in two *L. salmonis* strains differing in susceptibility to delousing agents. The study further assessed the effects of sublethal exposure to two salmon delousing agents, the pyrethroid DTM and the macrocyclic lactone EMB, on CaE transcript expression.

## 2 Materials and methods

### 2.1 Ethics statement

All research projects involving the University of Stirling (UoS) are subject to a thorough Ethical Review Process prior to any work being approved. The present research was assessed by the UoS Animal Welfare Ethical Review Body (AWERB) and passed the ethical review process. Laboratory infections of Atlantic salmon with *L. salmonis* were performed under a valid UK Home Office license and at low parasite densities unlikely to compromise fish welfare.

### 2.2 Identification of *L. salmonis* CaE genes

*L*. *salmonis* CaE homologues were identified by tBLASTn searches in *L. salmonis* transcriptome (EBI ENA reference ERS237607) and genome assemblies (LSalAtl2s, metazoan.ensembl.org), using *Drosophila melanogaster* CaEs (Oakeshott et al., 2005; Ranson et al., 2002) as queries (*E*-value cut-off = 10^−10^; minimum alignment length of 40 amino acids; Table S1). NCBI accession numbers for *D*. *melanogaster* CaEs are compiled in Table S2. Each identified putative CaE locus was manually annotated using BlastP searches against the “non-redundant” sequence collection from the NCBI.

### 2.3 Phylogenetic analyses

Phylogenetic analyses of *L. salmonis* CaEs further took into account CaEs of *D*. *melanogaster* and *Apis mellifera* (Claudianos et al., 2006) (NCBI accession numbers provided in Table S2). CaE amino acid sequences from *L. salmonis*, *D*. *melanogaster*, and *A*. *mellifera* and were aligned using default parameters in the online software MUSCLE version 3.8.31 (Multiple Sequence Comparison by Log-Expectation; https://www.ebi.ac.uk/Tools/msa/muscle/) (Edgar, 2004). Model selection using the likelihood-based Akaike Information Criterion was performed with the online software SMS: Smart Model Selection in PhyML version 3.3.20200621 (http://www.atgc-montpellier.fr/phyml-sms/) (Lefort et al., 2017). A maximum likelihood phylogenetic tree was constructed using RAxML version 8.0 (Stamatakis, 2014) with a WAG matrix plus optimized invariable sites (+I), gamma distributed rate heterogeneity among sites (+G), amino acid frequencies estimated from the data (+F), and 1000 bootstrap replicates. The phylogenetic tree was visualized with FigTree version 1.4.4.

### 2.4 Prediction of protein function and subcellular localization

*L*. *salmonis* CaE protein sequences were predicted from transcripts and analyzed using InterPro version 79.0 (ebi.ac.uk/interpro/), an integrated documentation resource covering databases for protein families, domains, and functional sites (Jones et al., 2014). Additional active site motifs were identified from an alignment of *L. salmonis* CaE amino acid sequences with *D*. *melanogaster* acetylcholinesterase (DmAChE) (NCBI accession no. 1QO9_A) using Clustal Omega version 2.1 (https://www.ebi.ac.uk/Tools/msa/clustalo/) (Sievers and Higgins, 2018). *L*. *salmonis* CaE sequences were predicted to encode catalytically competent enzymes if they contained the amino acid residues involved in the catalytic triad (Aranda et al., 2014), defined by serine, acidic (glutamate or aspartate) and histidine residues at positions corresponding to Ser238, Glu/Asp367, and His480 of the DmAChE sequence.

The program SignalP version 5.0 was used to predict putative signal peptide sequences of *L. salmonis* CaEs to identify proteins secreted by the secretory pathway (Almagro Armenteros et al., 2019). Subcellular localization of *L. salmonis* CaE proteins was assessed by DeepLoc version 1.0 (Almagro Armenteros et al., 2017).

### 2.5 *Lepeophtheirus salmonis* strains and husbandry

Laboratory *L. salmonis* strains used in this study have been described in detail elsewhere (Carmona-Antoñanzas et al., 2016; Heumann et al., 2012). Strain IoA-00, which was taken into culture in 2003, is susceptible to DTM, EMB, and azamethiphos. Strain IoA-02 was established in 2011 and is multi-resistant, with resistance levels based on acute bioassays being 143-fold for DTM, 4.3 to 7.3-fold for EMB, and 23-fold for azamethiphos (Carmona-Antoñanzas et al., 2017, Carmona-Antoñanzas et al., 2016; Humble et al., 2019).

*L*. *salmonis* strains were kept in culture at the Marine Environmental Research Laboratory of the University of Stirling (Machrihanish, UK). In brief, salmon lice were maintained on Atlantic salmon (*Salmo salar*, L.), which were held in circular tanks provided with a continuous supply of seawater and a photoperiod corresponding to natural day length. To propagate lines, egg strings obtained from gravid females were hatched and incubated to the infective copepodid stage, which were used to infect naïve Atlantic salmon. All laboratory infections were carried out under a valid UK Home Office license and at low parasite densities that were unlikely to compromise fish welfare. Infection trials were set up to produce preadult-II and adult parasites for chemical exposure experiments. Host fish were euthanized using a UK Home Office approved Schedule 1 method prior to the removal of salmon lice from fish.

### 2.6 Exposure of *L. salmonis* to deltamethrin and emamectin benzoate

*L*. *salmonis* adult males and preadult-II females of the drug susceptible strain IoA-00 and the multi-resistant strain IoA-02 were subjected to two concentrations of DTM (0.05 μg L^−1^ and 2 μg L^−1^) and EMB (25 and 150 μg L^−1^) to elucidate potential effects of sublethal drug treatments on CaE transcript abundance.

*L*. *salmonis* were collected from host fish as described above and allowed to recover for 2 to 6 h in aerated seawater at 12 °C. Individual parasites appearing viable based on attachment and swimming behavior were randomly allocated to 300 mL crystallizing dishes containing 100 mL of filtered (55 μm) seawater, with each dish receiving 5 preadult-II females and 5 adult males. Chemical exposures took place in a temperature-controlled chamber set to 12 °C. DTM and EMB were solubilized in PEG_300_ (polyethylene glycol, M_n_ = 300). Chemical exposures were initiated by adding 50 μL of a 2000× final concentration solution of the relevant compound to crystallizing dishes containing 100 mL seawater and salmon lice, resulting in a final solvent concentration of 0.05% (*v*/v) in all tests. No effects of PEG_300_ on transcript expression were detected in a previous microarray study (Carmichael et al., 2013).

Waterborne single exposures of *L. salmonis* involved a solvent control and two concentrations for each of the tested drugs (nominal concentrations: 0.05 μg L^−1^ and 2 μg L^−1^ DTM; 25 and 150 μg L^−1^ EMB). All drug treatments were expected to be sublethal to IoA-02, while the higher concentration of each drug was expected to be lethal to IoA-00 (Carmona-Antoñanzas et al., 2016; Heumann et al., 2012). In previous studies using the same bioassay methodology, measured drug concentrations in bioassays were 68 to 133% of nominal concentrations for DTM, and 50% of nominal concentrations for EMB (Carmichael et al., 2013; Carmona-Antoñanzas et al., 2017). Reflecting recommended conditions for *L. salmonis* bioassays (SEARCH Consortium, 2006), parasites were exposed to DTM for 30 min and then transferred to clean seawater for 24 h recovery, while exposures to EMB were for 24 h. Subsequently, the behavioral responses of test individuals were examined and rated. Rating criteria based on observed behavioral responses (live, weak, moribund, dead) have been described in detail elsewhere (Carmona-Antoñanzas et al., 2016). Parasites rated as “live” or “weak” were considered unaffected, while “moribund” and “dead” parasites were considered affected. Only individuals deemed unaffected were collected for RNA extraction and subsequent determination of transcript abundance. Parasites were sampled in RNA stabilization solution (4.54 M ammonium sulphate, 25 mM trisodium citrate, 20 mM EDTA, pH 5.4), stored overnight at 4 °C, and transferred to nuclease-free tubes for storage at −70 °C pending RNA extraction.

### 2.7 RNA extraction and cDNA synthesis

Individual salmon lice were homogenized in 1 mL TRI Reagent® (Sigma-Aldrich, Dorset, UK) using a bead-beater homogenizer (BioSpec, Bartlesville, Oklahoma, USA) and total RNA was extracted following the manufacturer's instructions. After phase separation, RNA was precipitated from the aqueous phase by adding 0.5 volumes of 2-propanol and 0.5 volumes of high salt buffer (0.8 M sodium citrate sesquihydrate; 1.2 M sodium chloride). Total RNA was resuspended in nuclease-free water (15 μL for adult males and 20 μL for preadult-II females). Quantity and quality of isolated total RNA were determined by UV spectrophotometry using a ND-1000 NanoDrop® (Thermo Scientific, UK) and RNA integrity was assessed by electrophoresis using 250 ng of denaturized total RNA in a 1% agarose gel stained with ethidium bromide. For each salmon louse, 2 μg total RNA was treated with 2 U DNase (DNA-*free*™ Kit, Ambion®) following the manufacturer's instructions. 2 μg DNA free total RNA of each sample were reverse transcribed using the High-Capacity cDNA Reverse Transcription Kit (Applied Biosystems, Warrington, UK) without RNase inhibitor, according to the manufacturers protocol. Reverse transcriptions were carried out including negative controls omitting RNA (NTC) and controls containing no enzyme (RT-). All cDNA samples were stored at −70 °C for further use.

### 2.8 Quantitative expression analysis by reverse transcription-quantitative PCR (RT-qPCR)

*L*. *salmonis* CaEs that contained an intact catalytic triad (see Section 2.4) and/or grouped into clades of high bootstrap support with *D*. *melanogaster*, *A*. *mellifera*, or *L. salmonis* CaE sequences with a conserved catalytic triad (see Section 2.3) were classified as potentially catalytically competent. As catalytically competent CaEs have the potential to mediate pesticide resistance by hydrolysis or sequestration, only potentially catalytically competent CaEs were selected for RT-qPCR studies. Six male and six female parasites were analyzed for each combination of treatment and strain. Five reference genes (ribosomal subunit 40S, *40S*; ribosomal subunit 60S, *60S*; elongation factor 1-alpha, *efa*; hypoxanthine-guanine phosphoribosyltransferase, *hgprt*; and RMD-5 homologue) were quantified and *40S* (M stability value = 0.244), *60S* (M stability value = 0.257), and *efa* (M stability value = 0.244) selected as reference genes as being most stable in *L. salmonis* according to GeNorm (Vandesompele et al., 2002).

The relative transcript expression of target and reference genes was measured by RT-qPCR using a Biometra TOptical Thermocycler (Analytik Jena, Goettingen, Germany) in 96-well plates. Primer sequences are provided in Table S3. Each sample was analyzed in duplicate 10 μL reaction volumes containing 5 μL Luminaris Colour Highreen qPCR Mix (Thermo Scientific, Hempstead, UK), 0.5 μL (10 pmol) each for the forward and reverse primer, 2.5 μL of 20-fold diluted cDNA for the target genes or 1 μL of 20-fold diluted cDNA for the reference genes and nuclease-free water. Each qPCR run was comprised of an activation step (50 °C for 2 min), then initial denaturation (95 °C for 15 min), followed by 35 cycles of denaturation, annealing, and extension (15 s at 95 °C, 30 s at the primer pair specific annealing temperature (Table S3), and 30 s at 72 °C). Finally, a melting curve with 1 °C increments during 6 s from 60 to 95 °C was performed to check the presence of a single product in each reaction. Control reactions included NTC and RT-.

For each RT-qPCR run, a standard curve was generated from a parallel set of reactions containing serial dilutions (1/5, 1/10, 1/20, 1/50, 1/100, 1/200, 1/500) of a cDNA pool derived from the samples. Standard curves were used to evaluate the efficiency of the primers, melting curves, and cycle threshold (Ct) values, and the combined efficiency of the primers and assay (Larionov et al., 2005). Primers used showed efficiencies in the range between 0.80 and 1.10 and resulted in amplifications characterized by a single melting peak and Ct values below 30. Ct values, melting curves, standard curves, and primer efficiencies were calculated by linked PCR cycler software (qPCR Soft 4.0). The size of the amplified qPCR product was checked by agarose gel electrophoresis along with appropriate markers and the reaction specificity was confirmed by sequencing the qPCR amplicon.

Relative transcript quantification was achieved by including on each PCR plate a parallel set of serial dilutions of a pool of all experimental cDNA samples, allowing derivation of the estimated relative copy number of the transcript of interest for each sample, corrected for the efficiency of the reaction. The normalised expression values (relative units, RUs) were generated by the ΔCt method (Pfaffl, 2001) with results expressed as the ratio between the estimated relative copy number of the target genes and a reference gene index calculated from the geometric mean of the estimated relative copy number of the three most stable reference genes *40S*, *60S* and *efa*.

### 2.9 Sequencing of *L. salmonis* CaE genes

*L*. *salmonis* CaE sequences that were predicted to be potentially catalytically competent were subjected to rapid amplification of 5′ and 3′ cDNA ends (RACE) to obtain their complete open-reading frame (Table S4). 5′ and 3′ RACE was carried out using the SMARTer RACE 5′/3′ Kit (Takara Bio, CA, USA) according to the manufacturer's protocol, using Q5® High-Fidelity 2× Master Mix (New England BioLabs Ltd., Hitchin, UK). under the following conditions: 98 °C for 30s, 5 cycles of 98 °C for 10 s and 72 °C for 1 min, then 5 cycles of 98 °C for 10s, 70 °C for 30s and 72 °C for 1 min, followed by 25 cycles of 98 °C for 10s, 68 °C for 30s and 72 °C for 1 min, and a final extension at 72 °C for 2 min. RACE products were separated by 1% agarose gel electrophoresis, purified and subcloned (pGEM-T Easy Vector system and *Escherichia coli* JM-109, Promega, WI, USA). Plasmids were isolated and inserts subjected to Sanger sequencing using a commercial service. The 5′ and 3′ amplicons and their associated CaE cDNA transcripts from the NCBI Nucleotide and EnsemblMetazoa databases were assembled using the software SeqMan Pro (DNASTAR, WI, USA). To confirm the assembly, each cDNA sequence was amplified in one PCR, subcloned, and sequenced (Table S4), as described above. Sequences obtained for the same PCR products were aligned to obtain contiguous cDNA sequences, which were deposed in the European Nucleotide Archive [project PRJEB40940] (see Table S4 for accession numbers).

### 2.10 Single nucleotide polymorphisms (SNPs) in CaE genes

To identify and analyze single nucleotide polymorphisms (SNPs) in CaE genes predicted to be catalytically competent, available RNA-seq data for strains IoA 00 and IoA-02 were used (ENA Project accession PRJEB41730). Using the hisat2 version 2.2.1 (Kim et al., 2019), sequencing reads were aligned to *L. salmonis* CaE cDNA sequences. Sequence variations were identified using the HaplotypeCaller function in GATK version 4.2.0.0 (Poplin et al., 2018).

### 2.11 Statistical analyses

Relative CaE expression data were tested for normality and homogeneity of variance using the Shapiro-Wilk's test and the Levene's test, respectively. As some data sets violated these homoscedasticity assumptions, non-parametric tests were employed in further analyses, performed in R version 3.5.0 (packages car, rcompanion, PMCMR). Effects of *L. salmonis* strain and sex/stage on CaE transcript expression were determined using the Scheirer-Ray-Hare test. The Kruskal-Wallis test was used to assess the effect of drug treatments on transcript expression. To account for the simultaneous testing of 10 transcripts and control the experiment-wise type I error, sequential Bonferroni correction was applied (Rice, 1989). After significant Kruskal-Wallis tests, Dunn's test was employed for post-hoc comparisons to the control group. Statistically significant expression differences between groups were considered biologically significant when exceeding the between-group difference of the estimated relative reference gene expression. In analyses of SNP expression between strains IoA-00 and IoA-02, genotype frequencies at each polymorphic site were compared using the Fisher's exact probability test, using the program Genepop version 4.7.5 (Raymond, 1995; Raymond and Rousset, 1995; Rousset, 2008). The significance level was set at *p* < 0.05 in all tests.

## 3 Results

### 3.1 Identification of *L. salmonis* CaEs

*L*. *salmonis* CaEs were identified by homology searches in a reference transcriptome (EBI ENA reference ERS237607) and a genome assembly (LSalAtl2s, metazoan.ensembl.org) of the species. Of a total of 21 putative CaE genes/pseudogenes identified in the genome, 20 had matching transcripts (Table S1), with three gene models being represented by more than one transcript. While 8 of the CaE sequences identified were partial, all *L. salmonis* CaE sequences lacked disabling frameshifts and in-frame stop codons.

### 3.2 Phylogenetic analyses and classification

**Fig. 1:**
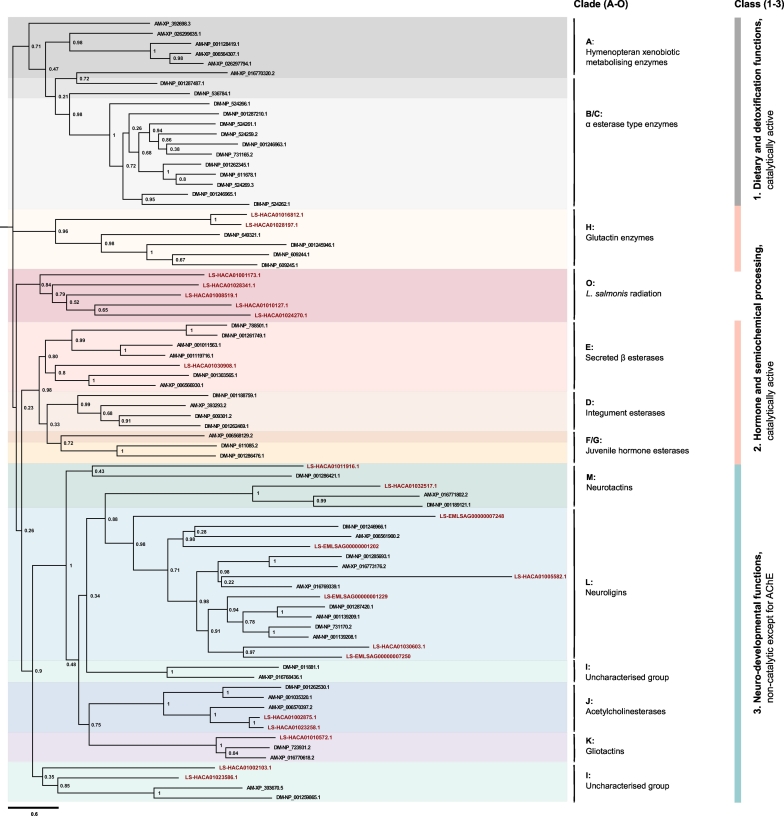
Phylogenetic relationship of carboxylesterases (CaEs) in *Lepeophtheirus salmonis*, *Drosophila melanogaster*, and *Apis mellifera*. The alignment was constructed using Multiple Sequence Comparison by Log-Expectation (MUSCLE) and phylogenetic relationship was conducted by Maximum likelihood (ML) analysis using RaxML. ML bootstrap support values (BS) (percentage of 1000 BS) are provided next to the nodes. *L*. *salmonis* (LS) CaEs are highlighted in red. DM *D*. *melanogaster*. AM: *A*. *mellifera*. (For interpretation of the references to colour in this figure legend, the reader is referred to the web version of this article.)

*L*. *salmonis* CaEs were subjected to phylogenetic analyses together with CaE sequences of *D*. *melanogaster* and *A*. *mellifera* (Fig. 1). The observed phylogenetic topology conforms to the phylogenetic classification scheme proposed by Oakeshott et al. (2005), who divided the CaE family into 14 clades (A-N) nested within three functional classes, with classes 1 to 3 being defined as the dietary/detoxification, the hormone/semiochemical processing, and the neuro/developmental classes, respectively. The 21 identified *L. salmonis* CaEs grouped into seven clades within two classes. The third class showed 13 *L*. *salmonis* members, which assigned to clades J (acetylcholinesterases (AChE); *n* = 2), K (gliotactins; *n* = 1), L (neuroligins; *n* = 6), M (neurotactins = 2), and I (uncharacterized proteins, n = 2), while the second class contained three members clustering into clades H (glutactins; n = 2) and E (secreted β esterases; n = 1). Five CaEs clustered together in a novel clade (clade O). BLAST annotation of *L. salmonis* CaEs confirmed the classification of sequences assigned to clades J to M as AChEs, gliotactins, neuroligins and neurotactins, respectively (Table S1). The two AChEs found in this study (**HACA01023258**.**1**, **HACA01002875**.**1**) have been described previously (Kaur et al., 2015a).

### 3.3 Conserved domains and predicted subcellular localization

**Fig. 2:**
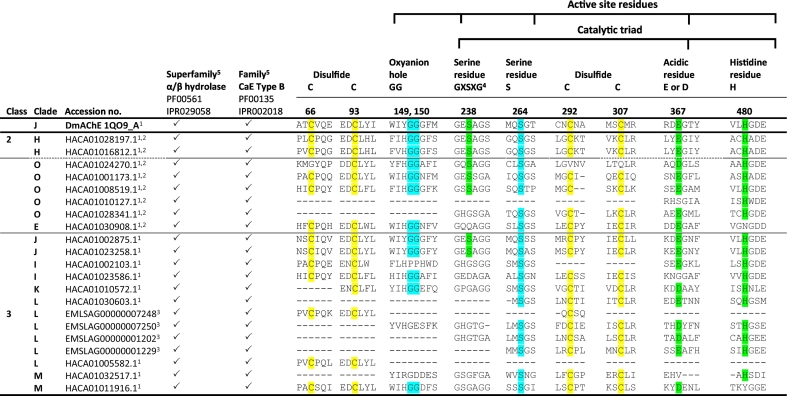
Conserved motifs in *L. salmonis* carboxylesterase (CaE) sequences. *L*. *salmonis* CaE sequences were aligned against the reference *Drosophila melanogaster* acetylcholine esterase (DmAChE) sequence. Amino acid residues were numbered according to DmAChE. Conserved catalytic triad residues (Ser238, Glu/Asp367, and His480) are shown in green. Additional conserved amino acid residues within the active site (oxyanion hole G149 and G150, putative catalytic tetrad residue Ser264 (Oakeshott et al., 2005)) are shown in blue. Conserved disulphide bridges (Cys66, Cys98 and Cys292, Cys307) are shown in yellow. “-” indicates a gap in the alignment. ^1^NCBI accession number. ^2^RT-PCR followed by Sanger sequencing was used to confirm cDNA sequences, which were deposited in the European Nucleotide Archive (see Table S5 for accession numbers). ^3^EnsemblMetazoa accession number. ^4^GXSXG: Nucleophilic elbow. ^5^Family affiliation according to Pfam (PF) and InterPro (IPR) entries. The carboxylesterase family type B belongs to the superfamily α/β hydrolase fold (PF00561, IPR029058). (For interpretation of the references to colour in this figure legend, the reader is referred to the web version of this article.)

*In-silico* analyses confirmed that the identified *L. salmonis* sequences were carboxylesterases possessing the Pfam PF00135 domain (Fig. 2) (Punta et al., 2012). Amino acid alignment of *L. salmonis* CaEs with *D*. *melanogaster* DmAChE revealed that seven *L. salmonis* sequences contained the amino acid motif of the catalytic triad, consisting of Ser, Glu or Asp and His residues, as well as amino acid residues constituting the active site, including the nucleophilic elbow (GXSXG), the oxyanion hole (GG), and a highly conserved Ser residue (Fig. 2). CaEs showing these features included all members of clade H within class 2, three members of the new clade O, and the two *L. salmonis* AChE (**HACA01023258**.**1**, **HACA01002875**.**1**) assigned to clade J in class 3. Three CaE sequences within clades O and E lacked catalytic triad residues but grouped in clusters of high bootstrap-support with *D*. *melanogaster*, *A*. *mellifera*, or *L. salmonis* CaE sequences with a conserved catalytic triad (Fig. 1, Fig. S1). Further bioinformatic analyses predicted members of clades K (gliotactins) and L (neuroligins) to be membrane associated (Table S5). In contrast, all members of the class 2 (clades H and E) were predicted to be soluble and secreted. Similarly, CaE sequences assigned to clade O were predicted to be soluble, possessing either a cytoplasmic or an endoplasmic reticulum targeting signal (Table S5).

### 3.4 Transcript expression of L. *salmonis* CaEs

Ten *L. salmonis* CaEs, which were predicted to be catalytically competent based on phylogenetic and protein functional analyses, were selected to study their transcript expression using qPCR.

The assessment of CaE transcript abundance in preadult-II females and adult males of the drug susceptible strain IoA-00 and the multi-resistant strain IoA-02 revealed significant effects of parasite sex/stage on transcript expression. As the estimated relative reference gene expression was found to be 2.14-fold larger in preadult-II females than in adult males (Table S6), only effects of sex/stage larger than 2.14-fold were considered biologically significant. Applying this threshold, five out of ten tested CaEs (**HACA01023258**.**1**, **HACA01030908**.**1**, **HACA01028197**.**1**, **HACA01016812**.**1**, **HACA01010127**.**1**) showed significant sex/stage-biased transcript expression (Table 1).

**Fig. 3:**
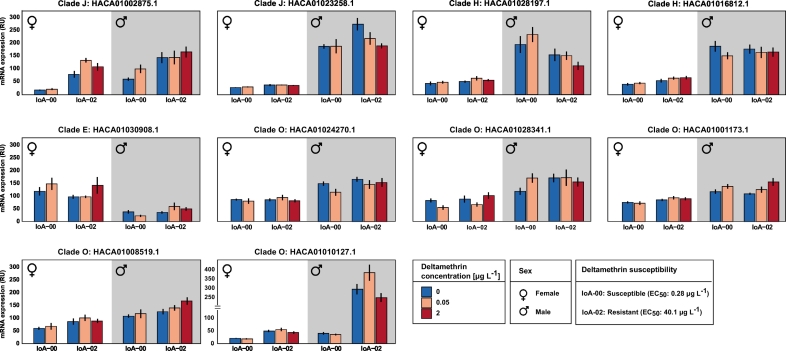
Effect of deltamethrin exposure on carboxylesterase (CaE) transcript expression in *L. salmonis*. Preadult-II females and adult males of the drug susceptible strain IoA-00 and the multi-resistant strain IoA-02 were exposed to deltamethrin (0.05 μg L^−1^; 2.0 μg L^−1^) for 30 min and allowed to recover for 24 h in seawater before esterase transcript expression was determined by quantitative reverse transcription polymerase chain reaction (RT-qPCR). Gene expression was expressed as relative units (RUs) calculated from the mean normalised ratios (*n* = 6 ± SE) between the estimated relative copy numbers of target genes and the estimated relative copy numbers of the reference genes. Bars bearing stars are significantly different (Dunn's test post-hoc comparisons to the control group).

**Fig. 4:**
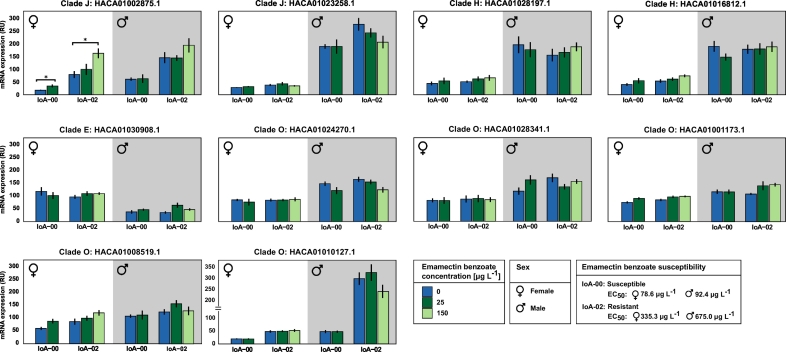
Effect of emamectin benzoate exposure on carboxylesterase (CaE) transcript expression in *L*. *salmonis*. Preadult-II females and adult males of the drug susceptible strain IoA-00 and the multi-resistant strain IoA-02 were exposed to deltamethrin (25 μg L^−1^; 150 μg L^−1^) for 30 min and allowed to recover for 24 h in seawater before esterase transcript expression was determined by quantitative reverse transcription polymerase chain reaction (RT-qPCR). Gene expression was expressed as relative units (RUs) calculated from the mean normalised ratios (n = 6 ± SE) between the estimated relative copy numbers of target genes and the estimated relative copy numbers of the reference genes. Bars bearing stars are significantly different (Dunn's test post-hoc comparisons to the control group); *significant at *p* < 0.05, **significant at *p* < 0.01.

In addition, transcript abundance of **HACA01002875.1** (clade J, *ace1b*) and **HACA01010127.1** (clade O) was significantly increased in strain IoA-02 compared to strain IoA-00 (*p* < 0.01) (Table 1). The effects of drug exposure were studied for the pyrethroid DTM (Fig. 3) and the macrocyclic lactone EMB (Fig. 4). Parasites of strains IoA-00 and IoA-02 were exposed to low sublethal concentrations of the compounds (0.05 μg L^−1^ DTM; 25 μg L^−1^ EMB), as well as higher concentrations (25 μg L^−1^ DTM; 150 μg l^−1^ EMB) that were tolerated by IoA-02 animals but lethal for IoA-00 parasites, with no survivors available for transcript expression studies (Table S7). Compared to transcript levels in untreated control parasites, transcript expression of **HACA01002875**.**1** (clade J, *ace1b*) was significantly increased (*p* < 0.05) in IoA-00 preadult-II females after treatment with 25 μg L^−1^ EMB and in IoA-02 preadult-II females after treatment with 150 μg L^−1^ EMB (Table 2).

**Table 1:** Carboxylesterase (CaE) transcript expression in two *L. salmonis* strains differing in drug susceptibility. Transcript expression of CaEs was determined by quantitative reverse transcription polymerase chain reaction (RT-qPCR) in preadult-II females and adult males of the drug susceptible strain IoA-00 and the multi-resistant strain IoA-02. Effects of strain, sex/stage, and interaction of strain and sex/stage were assessed by the Scheirer-Ray-Hare test.

Clade	NCBI accession no.	p-value Strain	Fold change Strain	p-value Sex/Stage	Fold change Sex/Stage	p-value Strain*Sex/Stage
J	HACA01002875.1	0.0011⁎⁎	2.84	0.012⁎	2.12	0.742
J	HACA01023258.1	0.094	1.44	0.0001⁎⁎⁎	7.0	0.905
E	HACA01030908.1	0.549	1.18	0.0001⁎⁎⁎	2.93	0.936
H	HACA01028197.1	0.908	1.16	0.0001⁎⁎⁎	3.66	0.564
H	HACA01016812.1	0.577	1.01	0.0001⁎⁎⁎	3.90	0.565
O	HACA01024270.1	0.805	1.07	0.0001⁎⁎⁎	1.85	0.613
O	HACA01010127.1	0.009⁎⁎	4.57	0.0001⁎⁎⁎	5.09	0.90
O	HACA01001173.1	0.644	1.00	0.0001⁎⁎⁎	1.41	0.488
O	HACA01028341.1	0.235	1.29	0.0023⁎⁎	1.70	0.332
O	HACA01008519.1	0.133	1.26	0.001⁎⁎⁎	1.60	0.686

⁎ Significant at *p* < 0.05.

⁎⁎ Significant at *p* < 0.01.

⁎⁎⁎ Significant at *p* < 0.001.

**Table 2:** Effect of chemical treatments on carboxylesterase (CaE) transcript expression in *L*. *salmonis*. Transcript expression of CaEs was determined by quantitative reverse transcription polymerase chain reaction (RT-qPCR) in preadult-II females and adult males of the drug susceptible strain IoA-00 and the multi-resistant strain IoA-02. Parasites were exposed to deltamethrin (0.05 μg L^−1^, 2.0 μg L^−1^) or emamectin benzoate (25 μg L^−1^, 150 μg L^−1^). For each strain, the CaE transcript expression was compared among chemical treatments and untreated controls using the Kruskal-Wallis test. The Dunn's test was employed for post-hoc comparisons of chemical treatments to the control group (see Fig. 2, Fig. 3). The experimental-wise type I error was controlled by sequential Bonferroni correction. CaEs that were significantly different expressed between a chemical treatment and the untreated control are shown in bold.

Clade	NCBI accession no.	*p*-value			
		Effect of chemical treatment			
		Female		Male	
		IoA-00	IoA-02	IoA-00	IoA-02
J	HACA01002875.1	**0**.**046**⁎	**0**.**027**⁎	0.066	0.590
J	HACA01023258.1	0.354	0.857	0.698	0.069
E	HACA01030908.1	0.224	0.571	0.051	0.170
H	HACA01028197.1	0.589	0.839	0.354	0.169
H	HACA01016812.1	0.557	0.284	0.227	0.924
O	HACA01024270.1	0.927	0.815	0.124	0.077
O	HACA01010127.1	0.543	0.703	0.593	0.069
O	HACA01001173.1	0.133	0.505	0.242	0.083
O	HACA01028341.1	0.162	0.348	0.066	0.531
O	HACA01008519.1	0.156	0.326	0.884	0.244

⁎ Significant at *p* < 0.05.

### 3.5 SNPs in CaE genes

Sequence variations in CaE genes that were predicted to be catalytically competent were identified by assessing RNAseq data available for individual male parasites of strains IoA-00 and IoA-02. Analyses revealed 15 SNP loci in five genes at which genotype frequencies differed significantly (*p* < 0.05) between the two strains (Table S8). Thirteen of these SNPs were missense mutations, i.e., encoded changes in the amino acid sequence, and 10 of these mutations occurred in proximity of the protein's active site (Fig. S2). Three SNPs within CaE genes **HACA01008519**.**1** (clade O; L374V and L375Q) and **HACA01023258**.**1** (*ace1a*; F362Y) corresponding to missense mutations were fixed in all tested individuals of the multi-drug resistant strain IoA-02 while absent in drug-susceptible IoA-00 strain parasites. Mutations L374V and L375Q are located in proximity to the catalytic triad of the polypeptide encoded by **HACA01008519**.**1**. The mutation F362Y in AChE1a has previously been described and was demonstrated to be associated with resistance towards the organophosphate azamethiphos (Kaur et al., 2015b).

## 4 Discussion

This study presents the first genome and transcriptome-wide survey of the CaE family in *L. salmonis*, which led to the identification of 21 genes/pseudogenes coding for CaEs. The present study further examined potential roles of CaEs in the resistance of *L. salmonis* to salmon delousing agents by comparing transcript expression of selected CaEs between a drug-susceptible and a multi-resistant strain of the parasite. Abundance of two CaE transcripts (**HACA01010127**.**1**, clade O; **HACA01002875**.**1**, clade J, *ace1b*) was significantly increased in a multi-resistant strain compared to a drug susceptible reference strain of the parasite. Moreover, expression of **HACA01002875**.**1** (*ace1b*) significantly increased (*p* < 0.05) in preadult-II females of both strains following exposure to sublethal concentrations of the macrocyclic lactone EMB.

In the present study, the CaE gene family in *L. salmonis* was annotated using the phylogenetic classification scheme proposed by Oakeshott et al. (2005), which divides the family into 14 clades (A-N) within three classes. Additional taxonomically informative characters for much of the phylogeny are the catalytic competence and the cellular/subcellular localization. The first dietary/detoxification class (clades A-C) contains catalytically competent enzymes with a wide range of cellular/subcellular localizations and comprises most CaEs involved in pesticide resistance in terrestrial arthropods. Members of the second hormone/semiochemical processing class (clades D—H) are catalytically competent, almost all secreted and, except for certain glutactins, not known to be membrane associated. In contrast, the third neuro/developmental class (clades J-M) contains mostly catalytically incompetent proteins that are generally membrane associated (Oakeshott et al., 2005). Based on their phylogenetic similarity and much of their predicted catalytic competence and subcellular localization, the *L. salmonis* CaE family can be partitioned into seven clades within two classes (Oakeshott et al., 2005).

None of the *L. salmonis* CaEs could clearly be assigned to the first class, known to possess detoxification functions (Oakeshott et al., 2005). In contrast, this class shows expansion in polyphagous or free-living ectoparasitic arthropods such as *D*. *melanogaster* (13 CaEs), *Tribolium castaneum* (26 CaEs), and *Anopheles gambiae* (16 CaEs), which presumably need to detoxify a wide variety of xenobiotics during their lifecycle (Table S9). Salmon lice only ingest host products when feeding and are partially protected from environmental toxicants during host-attachment. Thus, the absence of detoxifying first class CaEs in *L. salmonis* may have arisen from a reduced exposure to environmental toxins (Claudianos et al., 2006; Teese et al., 2010). Similarly, the human body louse *Pediculus humanus*, which is an obligate blood feeder, and *A*. *mellifera*, which maintains a mutualistic symbiotic relationship with flowering plants, possess only three and nine CaEs in the detoxifying class, respectively (Claudianos et al., 2006; Lee et al., 2010) (Table S9). Supporting this hypothesis, *L*. *salmonis* has been shown to possess a markedly reduced number of genes encoding detoxifying ABC transporters (*N* = 33) (Carmona-Antoñanzas et al., 2015) and CYPs (*N* = 25) (Humble et al., 2019), compared to *D*. *melanogaster* (56 ABC transporters and 85 CYPs) or *T*. *castaneum* (73 ABC transporters and 131 CYPs) (Broehan et al., 2013; Dean et al., 2001; Oakeshott et al., 2010).

Three *L. salmonis* CaEs were assigned to clades H (glutactins) and E (secreted β-esterases) within the second hormone/pheromone and semiochemical processing class. Both *L. salmonis* glutactins have a conserved catalytic triad. Similarly, eight *A*. *aegypti* glutactins (N_Total_ = 10) and one *D*. *melanogaster* glutactin (N_Total_ = 4) are predicted to be catalytically active, although their substrates remain to be identified (Oakeshott et al., 2005; Strode et al., 2008). *L*. *salmonis* has one member (**HACA01030908**.**1**) in clade E, containing characterized secreted β-esterase from *D*. *melanogaster* (**NP_001261749**.**1**, Est-6; **NP_788501**.**1**, Est-7) (Chertemps et al., 2012; Dumancic et al., 1997; Meikle et al., 1990) and *A*. *mellifera* (**NP_001011563**.**1**) (Claudianos et al., 2006; Kamikouchi et al., 2004). Moreover, **HACA01030908**.**1** encodes the *L. salmonis* CaE with the highest amino-acid similarity to validated β-esterases in *Popillia japonica* (**AAX58713**.**1**; Percent identity: 33.39%) (Ishida and Leal, 2008), *Antheraea polyphemus* (**AAX58711**.**1**; Percent identity: 30.95%) (Ishida and Leal, 2005; Vogt et al., 1985), and *Spodoptera littoralis* (**ACV60237**.**1**, Percent identity: 32.84%) (Durand et al., 2010). The above mentioned β-esterases have multiple functions, including metamorphic transition (**NP_788501**.**1**), reproductive functions (**NP_001261749**.**1**) (Meikle et al., 1990; Saad et al., 1994), degradation of plant odorants (Durand et al., 2010), and pheromone signaling (Est-6; **NP001011563**.**1**; **ACV60237**.**1**; **AAX58711**.**1**; **AAX58713**.**1**) (Chertemps et al., 2012; Ishida and Leal, 2008; Ishida and Leal, 2005; Durand et al., 2010). Like other arthropods, the putative *L. salmonis* β-esterase is predicted to be soluble and secreted. However, the sequence lacks conserved catalytic triad residues, which would most likely render it catalytically inactive. Interestingly, molecular work on *D*. *virilis* and *D*. *buzzatii* has also recovered secreted β-esterases that lack an intact catalytic triad (reviewed in Robin et al., 2009). However, their function remains to be identified, complicating functional predictions for the putative β-esterase in *L. salmonis*.

Most *L. salmonis* CaEs belong to the third neuro/developmental class, which comprises five out of seven shared clades between *L. salmonis*, insects, and chelicerates (Grbić et al., 2011). CaE genes are known to evolve rapidly, and the neuro/developmental class is the most ancient group. Accordingly, this class harbors the only overlapping radiations of vertebrate, *C*. *elegans*, and arthropod CaEs (clades J, K, L) (Oakeshott et al., 2005, Oakeshott et al., 1999). Except for AChE (J), all *L. salmonis* proteins within this class have an altered catalytic triad, indicating their hydrolytic inactivity. Based on the phylogenetic classification they are predicted to be involved in neurodevelopmental signaling and cell adhesion, i.e. neuroligins (clade L) have been implicated in synaptic growth, postsynaptic differentiation (Banovic et al., 2010; Sun et al., 2011), and sensory modulation (Biswas et al., 2010), neurotactins (clade M) have been characterized as being important for axon outgrowth, fasciculation, and guidance (Speicher et al., 1998), and gliotactins (clade K) have been shown to be responsible for septate junction formation (Genova and Fehon, 2003; Schulte et al., 2003) and the integrity of the transepithelial nerve-hemolymph permeability barrier (Auld et al., 1995).

The *L. salmonis* CaE family also comprises a new clade (clade O; five members), which could be found neither in the chelicerate *T*. *urticae* nor in insects (Tables S9, S10). As explained above, CaEs are known to evolve rapidly. Thus, this CaE lineage may has evolved after the separation of the subphyla Crustacea and Hexapoda in the Cambrian (~525 million years ago) (Giribet and Edgecombe, 2019). Similarly, the CaE gene family of the chelicerate *T*. *urticae* comprises two clades that are absent in both crustaceans and insects and may have evolved after the separation of the chelicerata and mandibulata in the ediacaran (~550 million years ago) (Grbić et al., 2011) (Table S9).

The present study identified seven *L. salmonis* CaEs that contained an intact catalytic triad and three CaEs that grouped into clades of high bootstrap support with *D*. *melanogaster*, *A*. *mellifera*, or *L. salmonis* CaE sequences with a conserved catalytic triad. The transcript expression of these ten CaEs was characterized in two L. *salmonis* strains differing in drug susceptibility and following sublethal exposure to DTM and EMB. Five out of ten tested CaEs showed significant sex/stage-biased transcript expression, with four transcripts being overexpressed by males. Sex-specific transcription of CaEs has previously been described in *L. salmonis* (Poley et al., 2016a) and other arthropod species. For example, male-biased expression of CaE transcripts within the seminal fluid of *D*. *melanogaster* has been shown to affect physiological processes in females when transferred during mating (Richmond et al., 1980). Moreover, specific odorant degrading CaE transcripts overexpressed in males were found to play a role in refreshing the sensory system to continually respond to chemosensory signals such as female sex-pheromones (Chertemps et al., 2012). Sex-specific CaE transcript expression has also been linked to sexual dimorphisms in morphology or feeding pattern (Poley et al., 2016b). In addition, CaEs can show developmental-specific expressions (Campbell et al., 2003). In the present study preadult-II female and adult male parasites were studied, so that the factors sex and stage are confounded, complicating the interpretation of CaE expression differences. Due to sex differences in *L. salmonis* size and development, the female preadult-II and male adult stages appear approximately at the same time in synchronized cohorts and are similar in size. Using these stages in this study ensured that all test animals experienced similar environmental conditions. Moreover, adult females of *L. salmonis* show significant within-stage growth and undergo cycles of oocyte production and vitellogenesis (Eichner et al., 2008), making this stage physiologically heterogeneous.

In the present study, expression of *ace1b* (**HACA01002875**.**1**, clade J) was significantly increased in multi-resistant IoA-02 salmon lice compared to drug susceptible IoA-00 parasites. The present study identified two *ace1* paralogues (*ace1a and ace1b*) in *L*. *salmonis*, confirming the findings of Kaur et al. (2015a). While AChE1a is predicted to be membrane bound, presumed to play the major role in cholinergic synaptic transmission, and the primary target for organophosphates, the physiological functions of AChE1b remain to be elucidated (Kaur et al., 2015a, Kaur et al., 2015b). The present study predicts that AChE1b is soluble. In *A*. *mellifera* and *D*. *melanogaster*, soluble AChEs have been suggested to play a non-neuronal role of chemical defense as bioscavenger, thereby providing protection against pesticides before they arrive at their target sites (Kim et al., 2014, Kim et al., 2012; Lee et al., 2015). Accordingly, upregulation of *ace1b* in the multi-resistant strain IoA-02 compared to the drug susceptible strain IoA-00 may contribute to drug resistance by sequestration or hydrolysis. In the present study, exposure to EMB caused significant upregulation of *ace1b* in females from strains IoA-00 and IoA-02. Soluble AChEs have also been shown to be overproduced in response to various stressors, including oxidative damage, psychological, physical, and chemical stressors (Birikh et al., 2002; García-Ayllón et al., 2012; Grisaru et al., 1999; Härtl et al., 2011; Lev-Lehman et al., 2000; Meshorer et al., 2002; Zimmerman and Soreq, 2006). Avermectins, which include EMB, are chemical stressors and have been shown to induce oxidative stress and DNA damage in crustaceans (Huang et al., 2019). As preadult-II female salmon lice have been found to be significantly more susceptible to EMB than adult males (Carmona-Antoñanzas et al., 2016; Poley et al., 2015), the upregulation of *ace1b* in females may be a response to EMB induced stress.

In the present study, expression of **HACA01010127**.**1** (clade O) was significantly increased in multi-resistant IoA-02 salmon lice compared to drug susceptible IoA-00 parasites. Based on its phylogenetic classification and cytosolic localization, **HACA01010127**.**1** is most closely related to cytoplasmic/intracellular proteins with dietary and/or detoxification functions (Oakeshott et al., 2005). However, RACE sequencing of **HACA01010127**.**1** revealed an altered catalytic triad, which would most likely render it catalytically inactive. To our knowledge, catalytically inactive proteins are not known to confer drug resistance.

In the present study, effects of drug exposures on CaE transcript expression were relatively moderate when determined at one time point after exposure. As gene induction can be a temporary event the experimental design may have failed to detect differential CaEs expression at earlier time points (Terriere, 1984). For example, in *M*. *domestica* time-dependent inductive expression patterns of CaEs have been observed within 12 to 72 h after permethrin challenge (Feng et al., 2018). Similarly, in *Plutella xylostella* pyrethroid exposure induced time-dependent alterations of carboxylesterase-6 mRNA expression levels within 3 to 48 h (Li et al., 2021). The design of exposure experiments in this report was aligned to recommendations for internationally standardized sea louse bioassays with DTM and EMB (Marín et al., 2018; Sevatdal and Horsberg, 2003; Westcott et al., 2008), allowing to compare results to those of other reports. In addition, in a previous study short EMB exposures (1−3 h) resulted in very few transcripts being up- or down regulated (Carmichael et al., 2013). The experiment described in the present manuscript has been previously analyzed with regards to drug exposure effects on CYP transcript expression, which was affected significantly by both DTM and EBM in expression were found (Humble et al., 2019).

In addition to pesticide resistance mechanisms involving an enhanced expression of CaEs (Field and Foster, 2002; Wei et al., 2019), resistance may alternatively be conferred by point mutations of CaE genes altering enzyme specificity and/or activity. For example, single nucleotide substitutions in α-esterases leading to amino acid replacements in the catalytic center have been shown to result in a loss of CaE activity and the acquisition of organophosphate hydrolase activity (Campbell et al., 1998; Claudianos et al., 1999; Newcomb et al., 1997). Furthermore, in *L. cuprina* mutations within the active site of CaEs have been shown to enhance the hydrolytic activity for several synthetic pyrethroids (Devonshire et al., 2007; Heidari et al., 2005). In the present study, SNP analyses in CaE genes revealed that two genes contained non-synonymous mutations affecting amino acid residues near the active site gorge of the respective polypeptide, which were fixed in all sequenced individuals from the multi-drug resistant strain IoA-02 and absent in parasites from the drug-susceptible strain IoA-00. One of these mutations, F362Y in AChE1a, has previously been linked to organophosphate resistance in *L. salmonis* (Kaur et al., 2015b). The other two mutations occurred in **HACA01008519**.**1** within clade O. More research is required to assess whether the mutation in **HACA01008519**.**1** affect susceptibility of *L. salmonis* to salmon delousing agents.

The present study investigated the association of drug resistance with changes at the transcriptional level of CaEs. However, it is also conceivable that the enzymatic activity of CaEs have been altered by post-transcriptional and/or post-translational modifications. Following transcription, translation of CaE mRNAs can be regulated via modification of translation-initiation factors, regulatory protein complexes that recognize elements usually present in untranslated regions (UTRs) of the target mRNA, or micro RNAs (miRNAs) that hybridize to mRNA sequences located in the 3′ UTR (Gebauer and Hentze, 2004). In addition, CaE enzyme activity can be altered by post-translational modifications such as amino acid changes, addition of macromolecules, or glycosylation, which have been implicated in protein stability and folding, targeting and recognition (Nalivaeva and Turner, 2001; Taylor and Feyereisen, 1996). For example, in organophosphate resistant *N*. *lugens* extensive differential post-translational glycosylation of CaE protein Nl-EST1 is believed to influence its stability, resulting in a non-linear correlation between Nl-EST1 mRNA levels and esterase activity (Small and Hemingway, 2000a, Small and Hemingway, 2000b; Vontas et al., 2000). Another study suggested an association between organophosphate resistance in Australian cattle tick (*R*. *microplus*) strains and post-translational modifications producing a drug-insensitive AChE (Baxter and Barker, 2002, Baxter and Barker, 1998).

Taken together, results from the present study suggest the potential involvement of *ace1b* (**HACA01002875**.**1**) in drug resistance in *L. salmonis*. However, it remains to be elucidated whether overexpression of *ace1b* is linked to DTM, EMB, and/or organophosphate resistance. No clear evidence was found for a role of other CaE genes in mediating resistance to EMB or DTM. Carmichael et al., 2013 found that expression of **HACA01002103**.**1** (clade I; referred to as **NP_001136104**.**1**) was moderately enhanced in EMB resistant salmon lice compared to a susceptible reference strain but, as shown in the present study, no significant differences in expression were apparent between susceptible and resistant salmon lice following EMB exposure. Similarly, no evidence has been found for a role of CYP genes in mediating EMB resistance (Humble et al., 2019). Thus, the genes under selection of EMB resistance in *L. salmonis* remain to be identified. For example, it has been suggested that EMB resistance involves differential gene expression of P-glycoprotein (Heumann et al., 2012; Igboeli et al., 2012), GABA-gated chloride channels (Carmichael et al., 2013), and neuronal acetylcholine receptors (Carmichael et al., 2013; Poley et al., 2015). Similar to EMB resistance, the present study provides no clear evidence for a role of CaE genes in mediating pyrethroid resistance, which is in line with studies by Poley et al. (2016a) and Sevatdal et al. (2005).

## 5 Conclusion

The CaE gene family of *L. salmonis* is one of the smallest characterized in arthropods to date. It includes catalytically inactive genes predicted to be involved in neurodevelopmental function, as well as secreted catalytically competent genes. In addition, the *L. salmonis* CaE gene family contains a new clade, which is predicted to be largely catalytically competent and soluble. Results from the present study suggest an association of overexpression of *ace1b* (**HACA01002875**.**1**) with drug resistance in *L. salmonis*. No clear evidence was found for a role of other CaE genes in mediating resistance to EMB or DTM.

The following are the supplementary data related to this article.

Supplementary material Image 1

Table S6 Cycle threshold (Ct) values and estimated relative copy numbers of the three most stable reference genes (ribosomal subunit 40S, 40S; ribosomal subunit 60S, 60S; elongation factor 1-alpha, efa) and ten carboxylesterase (CaE) transcripts in *L. salmonis*. Table S6

## Declaration of competing interest

The authors declare that they have no known competing financial interests or personal relationships that could have appeared to influence the work reported in this paper.
