# Supplementary material for: Genomic analysis of the carboxylesterase family in the salmon louse (Lepeophtheirus salmonis)
Source: Comp Biochem Physiol C Toxicol Pharmacol. 2021 Oct;248:109095. doi: 10.1016/j.cbpc.2021.109095 (PMC8387733; doi:10.1016/j.cbpc.2021.109095)
Supplement: Supplementary file 1 — Supplementary material [file mmc1.docx]

**Supplementary Material**

| Clade | Species | NCBI  accession no. | Superfamily  α/β hydro-lase^1^  PF00561 | Family^1^  PF00135 |  | |  |  |  |  |  |  |  |
| --- | --- | --- | --- | --- | --- | --- | --- | --- | --- | --- | --- | --- | --- |
|  |  |  |  |  | **Disulfide** | | **Oxyanion hole**  **GG** | **Serine residue**  **Catalytic triad**  **GXSXG**^2^ | **Serine residue**  **S** | **Disulfide** | | **Acidic**  **residue**  **E or D** | **Histidine residue**  **H** |
|  |  |  |  |  | **C** | **C** |  |  |  | **C** | **C** |  |  |
|  |  |  |  |  | 66 | 93 | 149, 150 | 238 | 264 | 292 | 307 | 367 | 480 |
| J | *D. melanogaster* | DmAChE 1QO9_A | 🗸 | 🗸 | ATCVQE | EDCLYI | WIYGGGFM | GESAGS | MQSGT | CNCNA | MSCMR | RDEGTY | VLHGDE |
| E | *D. melanogaster* | NP_788501.1 | 🗸 | 🗸 | VECMQW | EDCLTV | LLHGGAFM | GHSAGG | SVSGN | VGCGH | KDCLK | TEDGGY | TVHGDD |
| E | *D. melanogaster* | NP_001261749.1 | 🗸 | 🗸 | VACLQW | EDCLTV | HIHGGAFM | GHSAGG | SFSGN | VGCES | KKCLK | TEDGGY | TVHGDD |
| E | *A. mellifera* | NP_001303565.1 | 🗸 | 🗸 | PICLQR | EDCLYL | WFHGGGWQ | GESAGG | AQSGT | VGCGN | LECLR | AEEGLL | ACHAEE |
| E | *D. melanogaster* | NP_001011563.1 | 🗸 | 🗸 | FPCLQY | EDCLYL | WIHGGAFQ | GLSAGG | SISGT | MGCPT | IRCLR | SEEGLY | VCHADD |
| E | *A. mellifera* | NP_001119716.1 | 🗸 | 🗸 | SVCMQY | EDCLYI | WIHGGAFQ | GMSAGG | SISGV | MKCRT | IDCLQ | SKEGLY | VCHGDD |
| E | *A. mellifera* | XP_006566930.1 | 🗸 | 🗸 | NICVQR | EDCLYL | WFHGCGWI | GESAGG | SQSGN | LGCPS | VDCLR | SQEGSL | VSHADE |
| E | ***L. salmonis*** | **HACA01030908.1^3^** | **🗸** | **🗸** | **HFCPQH** | **EDCLWL** | **WIHGGNFV** | **GQQAGG** | **SLSGS** | **LECPY** | **IECIR** | **DDEGAF** | **VGNGDD** |

**Figure S1 Conserved carboxylesterase (CaE) motifs in Apis mellifera, Drosophila melanogaster, and Lepeophtheirus salmonis CaE sequences from clade E.** CaE sequences were aligned against the reference Drosophila melanogaster acetylcholine esterase (DmAChE) sequence. Amino acid residues were numbered according to DmAChE. Conserved catalytic triad residues (Ser238, Glu/Asp367, and His480) are shown in green. Additional conserved amino acid residues within the active site (oxyanion hole G149 and G150, putative catalytic tetrad residue Ser264 (Thomas et al., 1999)) are shown in blue. Conserved disulphide bridges (Cys66, Cys98 and Cys292, Cys307) are shown in yellow. “-“ indicates a gap in the alignment. ^1^Typology according to Pfam (PF) entries. ^2^Nucleophilic elbow. ^3^RT-PCR followed by Sanger sequencing was used to confirm cDNA sequences, which were deposited in the European Nucleotide Archive (See Table S4 for accession numbers).

**P1 subsite (leaving pocket)**

| Clade | NCBI accession no. | **Oxyanion hole** | | |  | | **Catalytic triad** | **P2 subsite (acyl pocket)** |  |  |  |  |  |  |  |
| --- | --- | --- | --- | --- | --- | --- | --- | --- | --- | --- | --- | --- | --- | --- | --- |
|  |  |  |  |  |  |  |  |  |  |  |  |  |  |  |  |
|  |  | **Changed AA within CaE gene** | | | **Gly, Gly** | **variable residues** | **Ser** | **variable residues** | **variable residues** | **variable residues** | **Glu or Asp** | **variable residues** | **variable residues** | **variable residues** | **His** |
| J | **DmAChE 1QO9_A** |  | | | **149, 150** | **162** | **238** | **271** | **328** | **330** | **367** | **370** | **371** | **440** | **480** |
| O | **HACA01008519.1** | P | **95** | P | 132, 133 | 143 | 212 | 247 | 300 | 302 | 344 | 347 | 348 | 423 | 466 |
|  |  | L | **374** | V |  |  |  |  |  |  |  |  |  |  |  |
|  |  | L | **375** | Q |  |  |  |  |  |  |  |  |  |  |  |
|  |  | E | **581** | * |  |  |  |  |  |  |  |  |  |  |  |
| O | **HACA01024270.1** | S | **148** | R | 108, 109 | 121 | 190 | 224 | 279 | 281 | 326 | 429 | 430 | 407 | 472 |
|  |  | F | **149** | S |  |  |  |  |  |  |  |  |  |  |  |
|  |  | Q | **150** | * |  |  |  |  |  |  |  |  |  |  |  |
|  |  | Q | **150** | R |  |  |  |  |  |  |  |  |  |  |  |
|  |  | S | **152** | P |  |  |  |  |  |  |  |  |  |  |  |
| 1A | **HACA01023258.1** | F | **362** | Y | 148, 149 | 161 | 230 | 263 | 319 | 321 | 358 | 361 | 362 | 432 | 472 |
|  |  | K | **431** | Q |  |  |  |  |  |  |  |  |  |  |  |
| 1B | **HACA01002875.1** | I | **88** | M | 148, 149 | 161 | 230 | 263 | 319 | 321 | 358 | 361 | 362 | 432 | 472 |
|  |  | S | **568** | P |  |  |  |  |  |  |  |  |  |  |  |
|  |  | V | **579** | E |  |  |  |  |  |  |  |  |  |  |  |
| H | **HACA01028197.1** | A | **338** | A | 254, 255 | 265 | 334 | 369 | - | 421 | 476 | 479 | 480 | 559 | 610 |

**Figure S2 Positions of mutated amino acids (AA) based on data shown in Table S8 and active site residues within carboxylesterase (CaE) genes.** L. salmonis CaE sequences were aligned against the reference Drosophila melanogaster acetylcholine esterase (DmAChE) sequence. Amino acid residues were numbered according to DmAChE. Catalytic triad residues are shown in green, residues of the oxyanion hole are shown in grey, residues of the P1 subsite (leaving pocket) are shown in blue, and residues of the P2 (acyl pocket) are shown in orange. Residues of the catalytic triad and the oxyanion hole show strong conservation across CaEs, while those constituting the P1 and P2 subsites are more variable. “-“ indicates a gap in the alignment. “*” indicates a stop codon.

**Table S1 The carboxylesterase (CaE) family in Lepeophtheirus salmonis.** CaEs were identified by homology searches in transcriptome (EBI ENA reference ERS237607) and genome assemblies (LSalAlt2s, ensemble.metazoa.org), using the entire complement of Drosophila melanogaster as queries. The assignment of sequences to classes and clades is based on the phylogenetic analysis shown in Figure 1.

| Annotation | | | |  |  |  |  |  |  |  |
| --- | --- | --- | --- | --- | --- | --- | --- | --- | --- | --- |
| Class | **Clade** | **Transcript annotation** | **Transcript accession no. (NCBI)** | | **Length (aa)** | **Gene accession no. (EnsemblMetazoa)** | **Best BLAST hit in D. melanogaster** | **NCBI accession number** | **E-value** | **Identity (%)** |
| 2 | **E** | Venom carboxylesterase 6 like | HACA01030908.1^4,6,7^ | | 647 | EMLSAG00000000224 | uncharacterized protein,  isoform B (CG6414) | NP_001303565.1 | 2.00E-84 | 34.8 |
|  | **H** | Putative protein | HACA01028197.1^6,7^ | | 742 | EMLSAG00000010508 | uncharacterized protein (CG9287) | NP_609244.1 | 5.00E-40 | 28.35 |
|  | **H** | Hypothetical protein | HACA01016812.1^6,7^ | | 695 | EMLSAG00000010605 | alpha esterase | AAB01153.1 | 8.00E-38 | 27.62 |
|  | **O** | Hypothetical protein | HACA01024270.1^6,7^ | | 583 | EMLSAG00000003802 | uncharacterized protein,  isoform B (CG6414) | NP_001303565.1 | 8.00E-55 | 28.95 |
|  | **O** | Putative esterase like | HACA01010127.1^4,6,7^ | | 302 | EMLSAG00000004652 | alpha-esterase | AAB01142.1 | 0.009 | 17.73 |
|  | **O** | Putative protein | HACA01001173.1^6,7^ | | 587 | EMLSAG00000008692 | uncharacterized protein,  isoform B (CG6414) | NP_001303565.1 | 3.00E-63 | 31.78 |
|  | **O** | Venom carboxylesterase 6 like | HACA01028341.1^4,6,7^ | | 410 | EMLSAG00000011806 | uncharacterized protein,  isoform B (G10175) | NP_732875.1 | 1.00E-28 | 27.96 |
|  | **O** | Juvenile hormone esterase like [Tribolium castaneum] | HACA01008519.1^4,6,7^ | | 589 | EMLSAG00000011990 | uncharacterized protein (CG4382) | NP_609301.2 | 7.00E-64 | 33.86 |
| 3 | **J** | Acetylcholinesterase 1B | HACA01002875.1^6^ | | 583 | EMLSAG00000002841 | acetylcholine esterase,  isoform C (CG17907) | NP_001262530.1 | 4.00E-128 | 36.25 |
|  | **J** | Acetylcholinesterase 1A | HACA01023258.1^6^ | | 630 | EMLSAG00000002842 | acetylcholine esterase,  isoform C (CG17907) | NP_001262530.1 | 1.00E-129 | 36.71 |
|  | **I** | CG4382 PA like [Tribolium castaneum] | HACA01002103.1^6^ | | 523 | EMLSAG00000004103 | uncharacterized protein (CG4382) | NP_609301.2 | 1.00E-42 | 28.04 |
|  | **I** | Esterase FE4 like  [Apis mellifera] | HACA01023586.1^6^ | | 556 | EMLSAG00000008264 | uncharacterized protein (CG3841) | NP_001188759.1 | 6.00E-74 | 33.93 |
|  | **K** | Gliotactin [Drosophila melanogaster] | HACA01010572.1^4,6^ | | 611 | EMLSAG00000008163 | gliotactin,  isoform A (CG3903) | NP_476602.1 | 0.00E+00 | 57.2 |
|  | **L** | Hypothetical protein | HACA01030603.1^4,5,6^ | | 697 | EMLSAG00000000872 | neuroligin 3,  isoform B (CG34127) | NP_001036685.2 | 3.00E-43 | 28.71 |
|  | **L** | Putative protein | HACA01001096.1 | | 744 | EMLSAG00000007250^1,4,6^ | neuroligin 3,  isoform B (CG34127) | NP_001036685.2 | 2.00E-94 | 33.04 |
|  | **L** | Putative protein | HACA01001097.1 | |  |  |  |  |  |  |
|  | **L** | Neuroligin 4 | HACA01001453.1 | | 761 | EMLSAG00000001202^2,6^ | neuoligin 1,  isoform E (CG31146) | NP_001246966.1 | 1.00E-57 | 33.18 |
|  | **L** | Neuroligin 4 | HACA01024283.1 | |  |  |  |  |  |  |
|  | **L** | Putative protein | HACA01025815.1 | | 772 | EMLSAG00000001229^3,6^ | neuroligin 3,  isoform B (CG34127) | NP_001036685.2 | 3.00E-60 | 33.82 |
|  | **L** | Neuroligin 1 like | HACA01027426.1^6^ | |  |  |  |  |  |  |
|  | **L** | Putative protein | HACA01030315.1^6^ | |  |  |  |  |  |  |
|  | **L** | Neuroligin 4 | HACA01004593.1^6^ | |  |  |  |  |  |  |
|  | **L** | Hypothetical protein | HACA01005582.1^4,6^ | | 138 | EMLSAG00000001231 | neuroligin 3,  isoform B (CG34127) | NP_001036685.2 | 2.00E-34 | 49.56 |
|  | **L** | - | - | | 147 | EMLSAG00000007248^4,6^ | neuroligin 4,  isoform C (CG34139) | NP_001036730.2 | 1.00E-25 | 57.14 |
|  | **M** | Neurotactin like [Tribolium castaneum] | HACA01032517.1^6^ | | 401 | EMLSAG00000003706 | neurotactin,  isoform C (CG9704) | NP_001189121.1 | 6.00E-65 | 35.41 |
|  | **M** | Putative protein | HACA01011916.1^6^ | | 648 | EMLSAG00000010413 | esterase 6,  isoform B (CG6917) | NP_001261749.1 | 6.00E-38 | 35.54 |

^1^Gene model EMLSAT00000007250 is the fusion between two transcript models, probably reflecting an assembly problem.

^2^Gene model EMLSAT00000001202 is the fusion between two transcript models, probably reflecting an assembly problem.

^3^Gene model EMLSAT00000001229 is the fusion between four transcript models, probably reflecting an assembly problem.

^4^Partial sequence only.

^5^Alternative splicing forms exist.

^6^Predicted polypeptide length based on this sequence.

^7^RT-PCR followed by Sanger sequencing was used to confirm cDNA sequences, which were deposited in the European Nucleotide Archive (see Table S5 for accession numbers).

**Table S2 The Apis mellifera and Drospophila melanogaster carboxylesterase (CaE) family.**

| Class | Clade | Species | NCBI accession no. | NCBI annotation | Superfamily α/β hydrolase fold^1^ PF00561/IPR029058 | Family CaE Type B^1^  PF00135/IPR002018 | Serine active site^1^  PS00122/IPR19826 |
| --- | --- | --- | --- | --- | --- | --- | --- |
| 1 | **A** | *Apis mellifera* | XP_392698.3 | esterase FE4 | 🗸 | 🗸 | 🗸 |
|  | **A** | *Apis mellifera* | XP_026299635.1 | bile salt-activated lipase isoform X2 | 🗸 | 🗸 | 🗸 |
|  | **A** | *Apis mellifera* | NP_001128419.1 | esterase A2 | 🗸 | 🗸 | 🗸 |
|  | **A** | *Apis mellifera* | XP_006564307.1 | carboxylesterase isoform X1 | 🗸 | 🗸 | 🗸 |
|  | **A** | *Apis mellifera* | XP_026297794.1 | esterase FE4 | 🗸 | 🗸 | 🗸 |
|  | **A** | *Apis mellifera* | XP_016770320.2 | esterase B1 | 🗸 | 🗸 | 🗸 |
|  | **B** | *Drosophila melanogaster* | NP_536784.1 | cricklet | 🗸 | 🗸 |  |
|  | **B** | *Drosophila melanogaster* | NP_001287487.1 | uncharacterized protein | 🗸 | 🗸 | 🗸 |
|  | **C** | *Drosophila melanogaster* | NP_524266.1 | alpha-Esterase-4 | 🗸 | 🗸 |  |
|  | **C** | *Drosophila melanogaster* | NP_001287210.1 | alpha-Esterase-3, isoform D | 🗸 | 🗸 |  |
|  | **C** | *Drosophila melanogaster* | NP_524262.1 | alpha-Esterase-6 | 🗸 | 🗸 |  |
|  | **C** | *Drosophila melanogaster* | NP_001246965.1 | alpha-Esterase-5, isoform B | 🗸 | 🗸 |  |
|  | **C** | *Drosophila melanogaster* | NP_001262345.1 | alpha-Esterase-2, isoform B | 🗸 | 🗸 |  |
|  | **C** | *Drosophila melanogaster* | NP_611678.1 | gasoline | 🗸 | 🗸 |  |
|  | **C** | *Drosophila melanogaster* | NP_524269.3 | alpha-Esterase-1 | 🗸 | 🗸 |  |
|  | **C** | *Drosophila melanogaster* | NP_524261.1 | alpha-Esterase-7 | 🗸 | 🗸 | 🗸 |
|  | **C** | *Drosophila melanogaster* | NP_001246963.1 | alpha-Esterase-10, isoform D | 🗸 | 🗸 | 🗸 |
|  | **C** | *Drosophila melanogaster* | NP_524259.2 | alpha-Esterase-8 | 🗸 | 🗸 | 🗸 |
|  | **C** | *Drosophila melanogaster* | NP_731165.2 | alpha-Esterase-9, isoform D | 🗸 | 🗸 | 🗸 |
| 2 | **D** | *Drosophila melanogaster* | NP_001188759.1 | uncharacterized protein | 🗸 | 🗸 | 🗸 |
|  | **D** | *Drosophila melanogaster* | NP_609301.2 | uncharacterized protein | 🗸 | 🗸 |  |
|  | **D** | *Drosophila melanogaster* | NP_001262469.1 | uncharacterized protein | 🗸 | 🗸 | 🗸 |
|  | **D** | *Apis mellifera* | XP_393293.2 | esterase E4 | 🗸 | 🗸 | 🗸 |
|  | **E** | *Drosophila melanogaster* | NP_788501.1 | esterase P | 🗸 | 🗸 | 🗸 |
|  | **E** | *Drosophila melanogaster* | NP_001261749.1 | esterase 6, isoform B | 🗸 | 🗸 | 🗸 |
|  | **E** | *Drosophila melanogaster* | NP_001303565.1 | uncharacterized protein | 🗸 | 🗸 | 🗸 |
|  | **E** | *Apis mellifera* | NP_001011563.1 | juvenile hormone esterase precursor | 🗸 | 🗸 | 🗸 |
|  | **E** | *Apis mellifera* | NP_001119716.1 | venom carboxylesterase-6 precursor | 🗸 | 🗸 | 🗸 |
|  | **E** | *Apis mellifera* | XP_006566930.1 | venom carboxylesterase-6-like | 🗸 | 🗸 | 🗸 |
|  | **F** | *Apis mellifera* | XP_006568129.2 | venom carboxylesterase-6-like | 🗸 | 🗸 |  |
|  | **G** | *Drosophila melanogaster* | NP_611085.2 | juvenile hormone esterase duplication | 🗸 | 🗸 | 🗸 |
|  | **G** | *Drosophila melanogaster* | NP_001286476.1 | juvenile hormone esterase, isoform C | 🗸 | 🗸 | 🗸 |
|  | **H** | *Drosophila melanogaster* | NP_649321.1 | esterase Q | 🗸 | 🗸 |  |
|  | **H** | *Drosophila melanogaster* | NP_001245946.1 | glutactin, isoform D | 🗸 | 🗸 |  |
|  | **H** | *Drosophila melanogaster* | NP_609244.1 | uncharacterized protein | 🗸 | 🗸 |  |
|  | **H** | *Drosophila melanogaster* | NP_609245.1 | uncharacterized protein | 🗸 | 🗸 |  |
| 3 | **J** | *Drosophila melanogaster* | NP_001262530.1 | acetylcholine esterase, isoform C | 🗸 | 🗸 | 🗸 |
|  | **J** | *Apis mellifera* | NP_001035320.1 | acetylcholinesterase 2 precursor | 🗸 | 🗸 | 🗸 |
|  | **J** | *Apis mellifera* | XP_006570397.2 | acetylcholinesterase | 🗸 | 🗸 | 🗸 |
|  | **I** | *Drosophila melanogaster* | NP_001259865.1 | uncharacterized protein | 🗸 | 🗸 |  |
|  | **I** | *Drosophila melanogaster* | NP_611881.1 | uncharacterized protein | 🗸 | 🗸 |  |
|  | **I** | *Apis mellifera* | XP_393670.5 | esterase E4 | 🗸 | 🗸 |  |
|  | **I** | *Apis mellifera* | XP_016768436.1 | carboxylesterase 5A | 🗸 | 🗸 | 🗸 |
|  | **K** | *Drosophila melanogaster* | NP_723931.2 | gliotactin, isoform F | 🗸 | 🗸 |  |
|  | **K** | *Apis mellifera* | XP_016770618.2 | neuroligin-4, Y-linked isoform X3 | 🗸 | 🗸 |  |
|  | **L** | *Drosophila melanogaster* | NP_001246966.1 | neuroligin 1, isoform E | 🗸 | 🗸 | 🗸 |
|  | **L** | *Drosophila melanogaster* | NP_001285693.1 | neuroligin 2, isoform C | 🗸 | 🗸 |  |
|  | **L** | *Drosophila melanogaster* | NP_731170.2 | neuroligin 3, isoform C | 🗸 | 🗸 |  |
|  | **L** | *Drosophila melanogaster* | NP_001287420.1 | neuroligin 4, isoform E | 🗸 | 🗸 |  |
|  | **L** | *Apis mellifera* | XP_006561900.2 | uncharacterized protein | 🗸 | 🗸 |  |
|  | **L** | *Apis mellifera* | XP_016773176.2 | neuroligin-1 isoform X3 | 🗸 | 🗸 |  |
|  | **L** | *Apis mellifera* | XP_016769339.1 | neuroligin 5 isoform X1 | 🗸 | 🗸 |  |
|  | **L** | *Apis mellifera* | NP_001139208.1 | neuroligin 3 precursor | 🗸 | 🗸 |  |
|  | **L** | *Apis mellifera* | NP_001139209.1 | neuroligin 4 precursor | 🗸 | 🗸 |  |
|  | **M** | *Drosophila melanogaster* | NP_001189121.1 | neurotactin, isoform C | 🗸 | 🗸 |  |
|  | **M** | *Drosophila melanogaster* | NP_001286421.1 | uncharacterized protein | 🗸 | 🗸 |  |
|  | **M** | *Apis mellifera* | XP_016771802.2 | neurotactin | 🗸 | 🗸 |  |

^1^Typology according to Pfam (PF), InterPro (IPR), and Prosite (PS) entries.

**Table S3 Oligonucleotide primer sequences used in quantitative reverse transcription polymerase chain reaction** **(RT-qPCR analyses).**

| Clade/Transcript annotation | NCBI accession no. | Forward primer (5' - 3') | Reverse primer (5' - 3') | Amplicon size (nt) | Efficacy (%) | Annealing tempera-ture (°C) |
| --- | --- | --- | --- | --- | --- | --- |
| Target genes | | | | | | |
| E | HACA01030908.1 | TCGAAGTAGACCAGATACGGAG | TTGGGCTCAGCATTTTCAGC | 102 | 86 | 60 |
| J, acetylcholinesterase 1B | HACA01002875.1^1^ | CATTGTCATGGAGTCTGGGATC | GGGAGGTTGTGCGTATGGTA | 144 | 87 | 60 |
| J, acetylcholinesterase 1A | HACA01023258.1^2^ | ACGCCAATTATTCTCGAGTTCTC | AGAACGTGGATGCAAAGGAC | 196 | 97 | 60 |
| H | HACA01028197.1 | AGATGTTGGGAAGTACGGGG | CGAAATTAAAGTCGCAGCAAGA | 141 | 86 | 60 |
| H | HACA01016812.1 | CGCACAATGGAGATACGATCA | CCATAGGGTTTCGGCAATTTC | 125 | 88 | 60 |
| O | HACA01024270.1 | CCTTCTTGTACCATGGATTCCC | TCTTCGTATCGTTTCCTCCAAG | 145 | 82 | 60 |
| O | HACA01010127.1 | GGAGGGGATGTCTTTTCTCTTTC | GACTGATGCCTGGACGTTTG | 104 | 96 | 60 |
| O | HACA01001173.1 | ACCTGATTTTCCATCTCAAGTGC | GGAGAGAAACCCATCGTGATC | 149 | 84 | 60 |
| O | HACA01028341.1 | GGGGTTGAGGATTGTCTTGTC | AGATCCACTCAACAATGCGTAG | 118 | 89 | 60 |
| O | HACA01008519.1 | TGCTAACTCTTCTCATGGTCC | GAAGCACCAAGATCCAGTCC | 94 | 84 | 60 |
| Reference genes | | | | | | |
| Ribosomal subunit 40S | BT121430.1 | AGTGTGGCCGGTGTTTAACAATCATCAA | GGGCTTCGAGTCCTTGTATGCTGCTGCTACT | 86 | 94 | 60 |
| Ribosomal subunit 60S | ACO10279.1 | CCTAGCTGCAATCACCATGA | CTCTTGCACTTGCTGCACTC | 197 | 87 | 55 |
| Elongation factor 1-alpha | EF490880.1 | CCAAATTAAGGAAAAGGTCGACAGACGTACTG | CAATGCCGGCATCACCAGACTTGA | 86 | 96 | 60 |
| Hypoxanthine‑guanine phosphoribosyltransferase | ACO14905.1 | GCAGCAAACATCGAATCTCA | TCTTTGCACGAACAAACTGC | 187 | 91 | 55 |
| RMD-5 homologue | ACO15319.1 | TCTCCTTATGCCCACTTGCT | GAGTTCCGTCCTTTGCATTC | 220 | 93 | 55 |

^1^Corresponding to NCBI nucleotide KJ132369.1.

^2^Corresponding to NCBI nucleotide KJ132368.1.

**Table S4 Oligonucleotide primer sequences used in rapid amplification of 3' and 5' cDNA ends (RACE) and subsequent sequencing of Lepeophtheirus salmonis cDNA sequence.**

| Clade | NCBI accession no. | Gen specific primer  3' RACE (5' - 3') | Gen specific primer  5' RACE (5' - 3') | Forward primer | Reverse primer | Additional sequencing primer 1 | Additional sequencing primer 2 | Additional sequencing primer 3 | RT-PCR product sequence ENA accession number |
| --- | --- | --- | --- | --- | --- | --- | --- | --- | --- |
| E | HACA01030908.1 | TGACGAAGGTGCTTTCAAAGGCTCTGC | GCCAAGTCGCGGGAAAATACATTGAGCC | GAACAAACGCAGATAAACACATC | TCATCCTTATCTTGAAGAGGTGG | AAAGCCCCTGTTCCCTTC | CCCTGGAGCGTGCAAAG | - | LR898355 |
| H | HACA01028197.1 | AGATGTTGGGAAGTACGGGGCATGCC | GTCCGTGACGTGAATAGGCCCATAATGT | GGGGAAAGTATGTGGGTTCG | TGACTGTGACTCCGCTTCTC | GAAACAACACGAGCCTGG | TGAAAGTTGGGAGTTGGC | - | LR898354 |
| H | HACA01016812.1 | TATATGATGTCCAAGCACGCCAGC | GCCAAGATCAAAGTGCTTCCTCCTTTCC | TCGCACAATGGAGATACGATC | AGCAGCAACATTCAAGGAAGG | GTTGGATGTGGACTTTGGC | TCCATGGGCCTTTGACAG | GGACGCAAATATTGAAAGCC | LR898353 |
| O | HACA01024270.1 | CCATGGAAGCCCTGTGTTGATGGTGG | GGAAGTACCGCCAAAGTGATGTATGTGG | GAGCTTGACCTTGGCCAATC | TTGTATTTCGAGCTTTCATCCAC | GGTGGAGCATTCATTCTTGG | TCTTACTCGCGTCTTGTCC | ATGGCTTTCAAGTGTACGG | LR898351 |
| O | HACA01010127.1 | CTGTTCATGGTGGATCGGCTCAGC | CCCCTAGAACAGTCCAATCCCTTCGTA | GTCGAGCAAGGGAAACATCC | GGCGAGGAGGAAACGGATAG | TGATGGGCCATGATGCAG | AACGTCACGACACTCTGG | - | LR898352 |
| O | HACA01001173.1 | TGGATTGCGGAAATGGGATGTATCGGTG | GACTCATTGGATAGCTGCAGGGACGC | CATGGGCAAAGATGTCTGAGA | GGTGATATCCGTGCTCAGTC | GAGAAGAGTACGCGAGGG | GGCAACATCACGCTTCATG | - | LR898356 |
| O | HACA01028341.1 | CCCAAGGGCCAACTATCCTACAGTCAAA | TTGACTGTAGGATAGTTGGCCCTTGGGT | ACCAAAGGAGATGTTCGTGG | AATTTCACCGGCTTCGTCTG | TTCTGGTGCTCATTCCGC | CCCAAGGGCCAACTATCC | - | LR898349 |
| O | HACA01008519.1 | ACAGATGTTCACTTCCTCGGACCCATC | ACATCTGCCTCATCGATTCCCATCAGG | ACAGTGTTTCTTGTAGTAGAGGA | CGACCTCTCTCTCCATGACAG | TCCCAAAGAAAGGAAGGTTC | GCAATGAGATGGGATGTGAC | GTTCACTTCCTCGGACCC | LR898350 |

**Table S5 Predicted subcellular localization and signal peptides of Lepeophtheirus salmonis carboxylesterase sequences.** Likelihood probability of subcellular localization predicted by DeepLoc-1.0, while signalP version 5.0 was used to predict putative signal peptide sequences.

| Class | Clade | Identifier | Type^1^  (Likelihood probability) | Signal peptide secretory pathway  Likelihood probability | Localization  (Likelihood probability) |
| --- | --- | --- | --- | --- | --- |
| 2 | **H** | HACA01028197.1^2,4^ | soluble (0.67) | 0.248 | Lysosome (0.427) |
|  | **H** | HACA01016812.1^2,4^ | soluble (0.947) | 0.953 | Endoplasmic reticulum (0.674) |
|  | **O** | HACA01024270.1^2,4^ | soluble (0.883) | 0.002 | Cytoplasm (0.371) |
|  | **O** | HACA01001173.1^2,4^ | soluble (0.952) | 0.069 | Endoplasmic reticulum (0.593) |
|  | **O** | HACA01008519.1^2,4^ | soluble (0.969) | 0.967 | Endoplasmic reticulum (0.746) |
|  | **O** | HACA01010127.1^2,4^ | soluble (0.916) | 0.0004 | Cytoplasm (0.682) |
|  | **O** | HACA01028341.1^2,4^ | soluble (0.698) | 0.001 | Cytoplasm (0.375) |
|  | **E** | HACA01030908.1^2,4^ | soluble (0.868) | 0.619 | Extracellular (0.347) |
| 3 | **J** | HACA01002875.1^2^ | soluble (0.864) | 0.325 | Endoplasmic reticulum (0.477) |
|  | **J** | HACA01023258.1^2^ | membrane (0.98) | 0.407 | Cell membrane (0.559) |
|  | **I** | HACA01002103.1^2^ | soluble (0.998) | 0.997 | Extracellular (0.894) |
|  | **I** | HACA01023586.1^2^ | soluble (0.965) | 0.988 | Endoplasmic reticulum (0.909) |
|  | **K** | HACA01010572.1^2^ | membrane (1) | 0.011 | Cell membrane (0.998) |
|  | **L** | HACA01030603.1^2^ | membrane (1) | 0.001 | Cell membrane (0.952) |
|  | **L** | EMLSAG00000007248^3^ | membrane (0.127) | 0.001 | Nucleus (0.132) |
|  | **L** | EMLSAG00000007250^3^ | membrane (1) | 0.005 | Cell membrane (0.973) |
|  | **L** | EMLSAG00000001202^3^ | membrane (1) | 0.011 | Cell membrane (0.935) |
|  | **L** | EMLSAG00000001229^3^ | membrane (1) | 0.004 | Cell membrane (0.999) |
|  | **L** | HACA01005582.1^2^ | membrane (0.281) | 0.001 | Mitochondrium (0.434) |
|  | **M** | HACA01032517.1^2^ | soluble (0.886) | 0.001 | Cytoplasm (0.252) |
|  | **M** | HACA01011916.1^2^ | soluble (0.963) | 0.547 | Endoplasmic reticulum (0.455) |

^1^Proteins were classified as membrane or soluble if they were found on either the membrane or the lumen of the organelle.

^2^NCBI Nucleotide accession number.

^3^EnsemblMetazoa accession number.

^4^RT-PCR followed by Sanger sequencing was used to confirm cDNA sequences, which were deposited in the European Nucleotide Archive (See Table S4 for accession numbers).

**Table S7 Rating of *L. salmonis* in bioassays with PEG300, emamectin benzoate, and deltamethrin.**

| Compound | Strain | Compound | Concentration | Female | | | Male | | | |
| --- | --- | --- | --- | --- | --- | --- | --- | --- | --- | --- |
|  |  |  |  | Live | Weak | Moribund | | Live | Weak | Moribund |
| PEG300 | IoA00 | PEG300 | 0.05% | 8 | 1 | 0 | | 9 | 0 | 0 |
| PEG300 | IoA02 | PEG300 | 0.05% | 8 | 0 | 1 | | 8 | 0 | 0 |
| EMB | IoA00 | EMB | 25 µg L^-1^ | 8 | 0 | 0 | | 8 | 0 | 0 |
| EMB | IoA02 | EMB | 25 µg L^-1^ | 7 | 1 | 0 | | 8 | 0 | 0 |
| EMB | IoA00 | EMB | 150 µg L^-1^ | 0 | 0 | 8 | | 0 | 0 | 8 |
| EMB | IoA02 | EMB | 150 µg L^-1^ | 8 | 0 | 0 | | 8 | 0 | 0 |
| DM | IoA00 | DTM | 0.05 µg L^-1^ | 8 | 0 | 1 | | 8 | 0 | 0 |
| DM | IoA02 | DTM | 0.05 µg L^-1^ | 7 | 0 | 1 | | 8 | 0 | 0 |
| DM | IoA00 | DTM | 2 µg L^-1^ | 0 | 0 | 9 | | 0 | 0 | 7 |
| DM | IoA02 | DTM | 2 µg L^-1^ | 8 | 0 | 0 | | 8 | 0 | 0 |

**Table S8 Single nucleotide polymorphism (SNP) loci within carboxylesterase (CaE) genes that showed significantly different genotype frequencies in two *L. salmonis*** **strains.** SNP analyzes in CaE genes were performed based on RNA-seq data of 15 adult male salmon lice of the drug susceptible strain IoA-00 and the multi-resistant strain IoA-02. P-values represent pairwise comparisons of genotype frequencies between both strains using the Fisher's exact probability test. Genotype frequencies were regarded significantly different between strains when the p≤0.05.

| Clade | NCBI accession no. | SNP locus | Allele 1 | Allele 2 | Strain | n | Genotypes (%) | | | Frequency allele 2 | Genotypic differentiation  Fisher's exact probability test | Corresponding  amino acid |
| --- | --- | --- | --- | --- | --- | --- | --- | --- | --- | --- | --- | --- |
|  |  |  |  |  |  |  | **gg** | **ga** | **aa** |  |  |  |
| O | **HACA01008519.1** | **358** | **A** | **G** | IoA-00 | 8 | 12.50 | 37.50 | 50.0 | 0.67 | 1.24E-02 | P95P |
|  |  |  |  |  | IoA-02 | 7 | 71.43 | 28.57 | 0.0 | 0.14 |  |  |
|  |  | **1193** | **C** | **G** | IoA-00 | 8 | 100.0 | 0.0 | 0.0 | 0.0 | 2.00E-04 | L374V |
|  |  |  |  |  | IoA-02 | 7 | 0.0 | 0.0 | 100.0 | 1.0 |  |  |
|  |  | **1197** | **T** | **A** | IoA-00 | 8 | 100.0 | 0.0 | 0.0 | 0.0 | 2.00E-04 | L375Q |
|  |  |  |  |  | IoA-02 | 7 | 0.0 | 0.0 | 100.0 | 1.0 |  |  |
|  |  | **1814** | **G** | **T** | IoA-00 | 8 | 100.0 | 0.0 | 0.0 | 0.0 | 2.00E-04 | E581* |
|  |  |  |  |  | IoA-02 | 7 | 0.0 | 100.0 | 0.0 | 0.5 |  |  |
| O | **HACA01024270.1** | **929** | **T** | **G** | IoA-00 | 8 | 100.0 | 0.0 | 0.0 | 0.0 | 1.30E-04 | S148R |
|  |  |  |  |  | IoA-02 | 7 | 0.00 | 85.71 | 14.29 | 0.57 |  |  |
|  |  | **931** | **T** | **C** | IoA-00 | 8 | 100.0 | 0.0 | 0.0 | 0.0 | 2.00E-04 | F149S |
|  |  |  |  |  | IoA-02 | 7 | 0.00 | 100.0 | 0.0 | 0.5 |  |  |
|  |  | **933** | **C** | **T** | IoA-00 | 8 | 100.0 | 0.0 | 0.0 | 0.0 | 2.00E-04 | Q150* |
|  |  |  |  |  | IoA-02 | 7 | 0.0 | 100.0 | 0.0 | 0.5 |  |  |
|  |  | **934** | **A** | **G** | IoA-00 | 8 | 100.0 | 0.0 | 0.0 | 0.0 | 2.00E-04 | Q150R |
|  |  |  |  |  | IoA-02 | 7 | 0.0 | 100.0 | 0.0 | 0.5 |  |  |
|  |  | **939** | **T** | **C** | IoA-00 | 8 | 100.0 | 0.0 | 0.0 | 0.0 | 2.00E-04 | S152P |
|  |  |  |  |  | IoA-02 | 7 | 0.0 | 100.0 | 0.0 | 0.5 |  |  |
| 1A | **HACA01023258.1** | **1086** | **T** | **A** | IoA-00 | 8 | 100.0 | 0.0 | 0.0 | 0 | 2.00E-04 | F362Y |
|  |  |  |  |  | IoA-02 | 7 | 0.0 | 0.00 | 100.0 | 1 |  |  |
|  |  | **1292** | **C** | **A** | IoA-00 | 6 | 33.33 | 50.00 | 16.67 | 0.417 | 2.25E-02 | K431Q |
|  |  |  |  |  | IoA-02 | 7 | 100.0 | 0.0 | 0.0 | 0 |  |  |
| 1B | **HACA01002875.1** | **308** | **G** | **T** | IoA-00 | 8 | 12.50 | 0.0 | 87.5 | 0.875 | 1.46E-03 | I88M |
|  |  |  |  |  | IoA-02 | 7 | 100.0 | 0.0 | 0.0 | 0 |  |  |
|  |  | **1746** | **T** | **C** | IoA-00 | 8 | 0.0 | 37.5 | 62.5 | 0.813 | 2.30E-04 | S568P |
|  |  |  |  |  | IoA-02 | 7 | 100.0 | 0.0 | 0.0 | 0 |  |  |
|  |  | **1780** | **T** | **A** | IoA-00 | 8 | 100.0 | 0.0 | 0.0 | 0 | 2.00E-04 | V579E |
|  |  |  |  |  | IoA-02 | 7 | 0.0 | 100.0 | 0.0 | 0.5 |  |  |
| H | **HACA01028197.1** | **1015** | **T** | **A** | IoA-00 | 8 | 75.0 | 25.0 | 0.0 | 0.125 | 4.10E-02 | A338A |
|  |  |  |  |  | IoA-02 | 7 | 14.29 | 85.71 | 0.0 | 0.429 |  |  |

* Stop codon.

**Table S9 Number of carboxylesterase (CaE) family members^1^ in eight arthropods.**

| Phylum | | Arthropods | | | | | | | | | |
| --- | --- | --- | --- | --- | --- | --- | --- | --- | --- | --- | --- |
| Clades | | Mandibulata - Altocrustacea | | | | | | | |  | **Chelicerata** |
| Subphylum | | **Hexapoda** | | | | | |  | **Crustacea** |  |  |
| (Sub)class | | Insecta | | | | | |  | Copepoda |  | Arachnida |
| Species | | ***A. mellifera*** | ***D. melanogaster*** | ***A. gambiae*** | ***B. mori*** | ***T. castaneum*** | ***P. humanus*** |  | ***L. salmonis*** |  | ***T. urticae*** |
| Dietary/detoxification class | |  |  |  |  |  |  |  |  |  |  |
| Clade A, B & C | | 8 | 13 | 16 | 55 | 26 | 3 |  | - |  | - |
| Hormone/semiochemical  processing class | |  |  |  |  |  |  |  |  |  |  |
| Clade D (Integument esterases) |  | 1 | 3 | - | 2 | 2 | - |  | - |  | - |
| Clade E (Secreted β esterases) | | 2 | 2 | 5 | 2 | 7 | 1 |  | 1 |  | - |
| Clade F & G (JHE) | | 2 | 3 | 9 | 4 | 2 | - |  | - |  | 2 |
| Clade H (Glutactins) | | 1 | 5 | 10 | 1 | 1 | 1 |  | 2 |  | 2 |
| Neuro/developmental class | |  |  |  |  |  |  |  |  |  |  |
| Clade I (Uncharacterized clade) | | 1 | 1 | 1 | 1 | 1 | 1 |  | 2 |  | - |
| Clade J (Acetylcholinesterases) | | 2 | 1 | 2 | 2 | 2 | 2 |  | 2 |  | 1 |
| Clade K (Gliotactins) | | 1 | 1 | 1 | 1 | 1 | 1 |  | 1 |  | 1 |
| Clade L (Neuroligins) | | 5 | 4 | 5 | 6 | 5 | 5 |  | 6 |  | 5 |
| Clade M (Neurotactins) | | 1 | 2 | 2 | 2 | 2 | 3 |  | 2 |  | 1 |
| Non-insect clades | |  |  |  |  |  |  |  |  |  |  |
| ^2^Clade O | | - | - | - | - | - | - |  | 5 |  | - |
| ^3^Clade J’ | | - | - | - | - | - | - |  | - |  | 34 |
| ^3^Clade J’’ | | - | - | - | - | - | - |  | - |  | 22 |
| ^3^undetermined | | - | - |  |  | - | - |  | - |  | 3 |
| Total | | **24** | **35** | **51** | **76** | **49** | **17** |  | **21** |  | **71** |

^1^Numbers from Claudianos et al. (2006), Oakeshott et al. (2005), Grbić et al. (2011), Yu et al. (2009), Lee et al. (2010), Oakeshott et al. (2010), and this study.

^2^Labelling according to this study.

^3^Labelling according to Grbić et al. (2011).

**Table S10 Summary of carboxylesterase (CaE) sequences within the new clade O in *Lepeophtheirus salmonis*.** CaEs were identified by homology searches in transcriptome (EBI ENA reference ERS237607) and genome assemblies (LSalAtl2s, ensemble.metazoa.org), using the entire complement of *Drosophila melanogaster* as queries. The assignment of sequences to clades is based on the phylogenetic analysis of *L. salmonis* CaEs shown in Fig. 1. The coding sequence of the *L. salmonis* CaEs within the new clade O were annotated using BLASTp searches against the NCBI Non-redundant Protein Sequence Collection of *Copepoda*, and aligned with *Tetranychus urticae* (class Arachnida) CaE sequences (Grbić et al., 2011).

| *Lepeophtheirus salmonis* | Annotation in *Copepoda* | | | | |  | Annotation in *Tetranychus urticae*^1^ | | | | | | |
| --- | --- | --- | --- | --- | --- | --- | --- | --- | --- | --- | --- | --- | --- |
| NCBI  accession no. | **BLAST hit** | **NCBI**  **accession no.** | **Species** | **E-value** | **Iden-tity (%)** |  | **Best BLAST hit** | ***T. urticae***  **gene ID** | **NCBI**  **accession no.** | **E-value** | **Iden-tity (%)** | ***T. urticae* clade** | |
| HACA01024270.1 | hypothetical protein TCAL_10027 | TRY70685.1 | *Tigriopus californicus* | 7.00  E-119 | 41.07% |  | Carboxylesterase 4A isoform X1 | tetur04g06380 | XP_015782259.1 | 1.00E-48 | 29.68% | | F' |
| HACA01001173.1 | Fatty acyl-CoA hydrolase precursor | XP_023334367.1 | *Eurytemora affinis* | 1E-85 | 32.62% |  | Esterase E4  isoform X1 | tetur23g00910 | XP_015790984.1 | 5.00E-61 | 33.21% | | F' |
| HACA01008519.1 | hypothetical protein TCAL_05343 | TRY76473.1 | *Tigriopus californicus* | 9.00  E-123 | 36.47% |  | Carboxylesterase 4A isoform X1 | tetur04g06380 | XP_015782259.1 | 3.00E-74 | 33.54% | | F' |
| HACA01010127.1 | hypothetical protein TCAL_02277 | TRY67019.1 | *Tigriopus californicus* | 7.00  E-25 | 31.62% |  | Carboxylesterase 4A isoform X1 | tetur04g06380 | XP_015782259.1 | 0.001 | 22.22% | | F' |
| HACA01028341.1 | hypothetical protein TCAL_11235 | TRY61787.1 | *Tigriopus californicus* | 1.00  E-40 | 28.83% |  | Esterase E4  isoform X1 | tetur23g00910 | XP_015790984.1 | 2.00E-20 | 26.79% | | F' |

**^1^**The *T. urticae* CaE gene family contains 71 genes with two new clades representing 34 (clade J’) and 22 (clade J’’) CaEs, respectively (Grbić et al., 2011).
